# Formation of a Polarised Primitive Endoderm Layer in Embryoid Bodies Requires Fgfr/Erk Signalling

**DOI:** 10.1371/journal.pone.0095434

**Published:** 2014-04-21

**Authors:** Gail Doughton, Jun Wei, Nicolas Tapon, Melanie J. Welham, Andrew D. Chalmers

**Affiliations:** 1 Department of Biology and Biochemistry and the Centre for Regenerative Medicine, University of Bath, Bath, United Kingdom; 2 Department of Pharmacy and Pharmacology and the Centre for Regenerative Medicine, University of Bath, Bath, United Kingdom; 3 Apoptosis and Proliferation Control Laboratory, Cancer Research UK, London Research Institute, London, United Kingdom; Wellcome Trust Centre for Stem Cell Research, United Kingdom

## Abstract

The primitive endoderm arises from the inner cell mass during mammalian pre-implantation development. It faces the blastocoel cavity and later gives rise to the extraembryonic parietal and visceral endoderm. Here, we investigate a key step in primitive endoderm development, the acquisition of apico-basolateral polarity and epithelial characteristics by the non-epithelial inner cell mass cells. Embryoid bodies, formed from mouse embryonic stem cells, were used as a model to study this transition. The outer cells of these embryoid bodies were found to gradually acquire the hallmarks of polarised epithelial cells and express markers of primitive endoderm cell fate. Fgf receptor/Erk signalling is known to be required for specification of the primitive endoderm, but its role in polarisation of this tissue is less well understood. To investigate the function of this pathway in the primitive endoderm, embryoid bodies were cultured in the presence of a small molecule inhibitor of Mek. This inhibitor caused a loss of expression of markers of primitive endoderm cell fate and maintenance of the pluripotency marker Nanog. In addition, a mislocalisation of apico-basolateral markers and disruption of the epithelial barrier, which normally blocks free diffusion across the epithelial cell layer, occurred. Two inhibitors of the Fgf receptor elicited similar phenotypes, suggesting that Fgf receptor signalling promotes Erk-mediated polarisation. This data shows that primitive endoderm cells of the outer layer of embryoid bodies gradually polarise, and formation of a polarised primitive endoderm layer requires the Fgf receptor/Erk signalling pathway.

## Introduction

A key cell fate decision in early mammalian development occurs when cells in the inner cell mass (ICM) decide to follow either the primitive endoderm or epiblast cell fate [1]. The primitive endoderm forms an epithelium which localises between the blastocoel cavity and the epiblast, these three components are surrounded by a second epithelium, the trophectoderm. Two subpopulations are formed from the primitive endoderm, the parietal and visceral endoderm give rise to the yolk sac, whilst the visceral endoderm also contributes to the gut endoderm and provides embryonic patterning signals [2]. In contrast, cells of the epiblast give rise to the embryo proper. Recently, there has been a rapid increase in our understanding of how the primitive endoderm versus epiblast cell fate decision is regulated (Reviewed in [2], [3]). These studies have used mouse embryos, as well as embryoid bodies which can be formed from mouse embryonic stem (mES) cells. The outer cell layer of embryoid bodies is an epithelium with many similarities to the embryonic primitive endoderm, and has therefore been used as a model of its development [4], [5].

Cell fate decisions in the ICM cells are a dynamic process: cells initially express both epiblast (e.g. Nanog) and primitive endoderm markers (e.g. Gata6 and Gata4), the expression then resolves into a salt-and-pepper pattern within the ICM, where cells express either epiblast or primitive endoderm markers [6]–[8]. The cells of the primitive endoderm then migrate to their final position facing the blastocoel cavity where they form an epithelial sheet.

Fgf receptor (Fgfr) signalling via the Raf/Mek/Erk signalling (Erk signalling) pathway has an important role in promoting primitive endoderm formation (Reviewed in [9]). For instance Grb2, an adaptor protein that links receptor tyrosine kinases to the Erk cascade, is essential for primitive endoderm development [10]. Interestingly, activated H-Ras expression in Grb2-deficient mES cells promotes endoderm differentiation in embryoid bodies, suggesting Grb2 functions through activation of Ras in this context [10]. A more detailed analysis of the Grb2^−/−^ mouse phenotype demonstrated that Gata6 expression is lost and all ICM cells are Nanog positive [6]. In addition, when embryoid bodies are formed from mES cells transfected with a constitutively active Mek mutant, Nanog expression is repressed, and primitive endoderm differentiation occurs [11]. This suggests that the Erk signalling pathway mediates Nanog repression, which is known to be required for differentiation of ICM cells into primitive endoderm. These results led to the hypothesis that the mosaic expression of epiblast and primitive endoderm markers is dependent upon a Grb2-Ras-Mek/Erk signalling cascade. Accordingly, Grb2^−/−^ ES cells transfected with Gata6 or Gata4 are induced to undergo endoderm differentiation, and are able to form primitive endoderm in embryoid bodies, arguing that Grb2-Erk signalling acts upstream of Gata6 and Gata4 to promote primitive endoderm cell fate [12].

Fgf4 activation of Fgfr appears to be the input responsible for activating the Erk signalling pathway to promote primitive endoderm cell fate [13]–[15]. Treatment of embryos with a combination of inhibitors for both the Fgfr and Mek causes ICM cells to express Nanog whilst repressing primitive endoderm markers Gata6 & 4 [16], [17]. Conversely, treatment of embryos with Fgf4 and heparin causes all cells of the ICM to become Gata6 positive [17], [18]. In Fgf4^−/−^ embryos Gata6 and Nanog colocalise until the 32-cell stage, but by the 64-cell stage only a small number of weakly Gata6 positive cells could be detected, whilst Gata4 was never detected. This mutant could not be rescued by addition of exogenous Fgf at a uniform concentration suggesting *in vivo* it is regional differences in Fgf concentration which produce the salt-and-pepper pattern [19]. Together, these studies provide convincing evidence that Fgf4/Fgfr activation of Erk signalling is essential for primitive endoderm specification.

In contrast to our growing understanding of cell fate specification less is known about the mechanisms which regulate polarisation of primitive endoderm cells. A number of studies have shown a polarised localisation of proteins within the epithelial cells of the primitive endoderm. E-cadherin is localised to their basolateral membrane [20], and Lrp2 localises to the apical surface of the primitive endoderm in E4.5 mouse embryos [21]. The adaptor protein Disabled-2 (Dab2) also localises apically and is required for the establishment of epithelial polarity [22], [23] and Laminin, a constituent of the basement membrane is required for proper epithelial organisation of the primitive endoderm [24].

In the trophectoderm epithelial polarisation has been tightly linked to the regulation of cell fate [25] and a role for polarity in the control of the primitive endoderm cell fate has also been suggested. One aspect to this is that epithelial polarity in the primitive endoderm cells appears to anchor the sorted cells to their final localisation on the outer surface of the epiblast [20], [22], [23]. If aPkc, a key regulator of cell polarity, is inhibited the primitive endoderm cells do not become anchored [26]. No effect was observed on early primitive endoderm markers (e.g. Pdgfra), but later there was a reduction in Gata4 positive cells. This suggests that in addition to being essential for sorting, aPkc is also required for maturation of the primitive endoderm cell fate [26]. Despite its well established role in promoting primitive endoderm fate, it is not clear if the Erk signalling pathway is required for formation of a polarised primitive endoderm.

Here, we use embryoid bodies as a model to investigate the mechanisms which promote polarisation of the primitive endoderm, and determine whether the Fgfr/Erk signalling pathway is required for polarisation of this tissue. We show that embryoid bodies gradually expressed markers of primitive endoderm cell fate and down regulate Nanog. Polarity complex, tight junction, adherens junction, and basement membrane proteins also developed a polarised localisation in the primitive endoderm layer. Treatment with small molecule inhibitors of Mek or the Fgfr caused the outer cells of the embryoid body to fail to upregulate primitive endoderm markers and no longer downregulate Nanog expression. Mislocalisation of the proteins which normally show apico-basolateral polarisation in the primitive endoderm cells also occurred. Additionally, disruption of the epithelial barrier, which normally blocks free diffusion across the epithelium of the primitive endoderm, was observed. These results show that Fgfr driven Erk signalling is required for the formation in embryoid bodies of a primitive endoderm layer which exhibits apico-basolateral polarity and epithelial barrier function.

## Methods

### Culture of mESCs

The E14tg2A mES cell line (Clone R63) was a kind gift of Dr Owen Witte, UCLA, California, [27]. mESCs were routinely cultured on dishes coated in 0.1% (w/v) porcine gelatin (Sigma) in Knock-out Dulbecco’s modified Eagles Medium (Gibco) supplemented with 15% (v/v) knockout serum replacement (Gibco), 2 mM L-Glutamine (Gibco), 0.1 mM 2-mercaptoethanol (Bio-Rad), 0.1 mM non-essential amino acids (Gibco) and 10^3^ units/ml murine Leukaemia inhibitory factor (LIF) (ESGRO; Chemicon: ESG1106). Occasionally, cells were cultured with 1i, addition of 2 µM 1m (GSK3β inhibitor (Bone et al. 2009)), to maintain the pluripotency of the cells.

For embryoid body formation, cells were tryspinised in 0.05% Trypsin-EDTA (Invitrogen) and resuspended at a density of 40,000 cells/ml in Dulbecco’s modified Eagles Medium (Invitrogen) supplemented with 20% serum (Invitrogen) and 25 mM 2-mercaptoethanol (Sigma). Small-molecule inhibitors were added to the cell suspension at concentrations indicated in main text; 0.5 µM-4 µM PD-0325901 (Axon MedChem: 1408), 1 µM-8 µM AZD-4547 (Santa Cruz: Sc-364421), 100 nM PD-173074 (Selleckchem: S1264). Control embryoid bodies were formed from media containing the same DMSO percentage as used with the inhibitors, this ranged from 0.02% to 0.08% (See figure legends for the concentration used in each experiment). 25 µl drops of the cell suspension were pipetted onto the inside of the lid of a 10 cm tissue culture dish. 10 mls of phosphate-buffered saline (PBS) (Invitrogen) was placed in the bottom of the plate. If cultured for longer than 5 days, on day 5 embryoid bodies were washed using PBS, put in fresh media and plated on a ultra-low adherent dish (Corning: 3471). Images of unstained embryoid bodies were taken on a Leica MZFLIII microscope.

### Cell Lysates

Lysates were prepared by washing embryoid bodies twice in PBS. Ice-cold RIPA buffer was added which contains 150 mM NaCl (Sigma), 50 mM Tris HCl pH 8 (Sigma), 1% NP-40 (VWR), 0.5% Na Deoxycolate (Fisons), 0.1% SDS (Sigma), 25 units/ml Benzonase nuclease (Sigma: E1014), supplemented with either phosphatase inhibitor cocktails 2 and 3 (Sigma: P5726 & P0044) and c*O*mplete Mini, EDTA-free protease inhibitor cocktail (Roche: 04693159001) or the following protease/phosphatase inhibitors: 1 mM sodium vanadate (Sigma: S-6508), 1 mM sodium molybdate (BDH AnalR: 10254), 10 mM sodium fluoride (Sigma: S6521), 10 µg/ml Phenylmethylsulphonyl fluoride (PMSF) (Sigma: P7626), 0.7 µg/ml Pepstatin A (Sigma: P5318), 10 µg/ml Aprotinin (Roche: 236624), 10 µg/ml Leupeptin (Sigma: L8511), 10 µg/ml Soyabean trypsin inhibitor (Roche: 109886) in H_2_O. Samples were kept at −20°C until required. Protein concentrations were determined using the BCA assay (Thermo Scientific) according to the manufacturer’s directions.

### Immunoblotting

Cell lysates were separated by SDS PAGE using 7.5 or 10% polyacrylamide gels using the Bio-Rad system or gradient Novex Bis-Tris Gels using an XCell SureLock Mini Cell (Invitrogen). Immunoblotting was performed using a semi-dry blotter (Amersham Biosciences, Multiphor II) or the Bio-Rad wet-transfer system, transfer buffer used contained 39 mM glycine, 48 mM Tris base, 0.00375% SDS, 20% (v/v) methanol. Nitrocellulose (GE) or PVDF membrane (GE) was incubated with primary antibodies overnight at 4°C, secondary antibody was added for 1 hour. Immunoblotting was carried out using primary antibodies at the following concentrations: 1∶500 mouse anti-ppErk (Sigma Aldrich: M9692) 1∶1000 rabbit anti-Total-Erk (Cell Signalling Technology: 4695S), goat anti-Gata6 (R&D systems: AF1700), rabbit anti-Gata4 (Santa-Cruz: sc-9053), rabbit anti-aPkcζ/λ (Santa-Cruz: sc-216), rabbit anti-Fibronectin (Sigma: F3648), 1∶5000 rabbit anti-β-catenin (Cell Signalling Technology 9562), mouse anti-Tubulin (Sigma: T9026) 1∶40,000 mouse anti-Gapdh (Ambion: 4300). Anti-rabbit, anti-mouse, and anti-goat secondary antibodies conjugated to horseradish peroxidase were used at 1∶10,000 (DAKO), or 1∶5000 (GE). Blots were developed using ECL prime (GE Healthcare) or ECL2 (Thermo Scientific) according to manufacturer’s directions. Detection was carried out using an ImageQuant RT ECL system or Amersham Hyperfilm ECL (GE Healthcare) with quantification using ImageJ (NIH). Nitrocellulose blots were stripped for 60 minutes at 55°C in stripping buffer (0.2 M Tris PH 6.7, 2% SDS, and 0.1 M β-mercaptoethanol), PVDF blots were stripped for 10 minutes at room temperature using 0.5 M NaOH. Blots were subsequently washed in TBST and then blocked. For statistical analysis a 1-way ANOVA with a Dunnetts post-hoc test was performed using GraphPad Prism 5.

### Immunofluorescence Staining

At the appropriate time point, embryoid bodies were fixed with 4% (w/v) paraformaldehyde fixation buffer (PFA) in PBS for 1 hour and permeabilised in 0.25% triton (Sigma) (vol/vol) in PBS for 20 minutes. Non-specific binding was blocked by 3% BSA (sigma) +0.1% tween (Sigma) in PBS for 30 minutes. Primary and secondary antibodies were diluted in 3% BSA (w/v) +0.1% tween (vol/vol) in PBS. Embryoid bodies were incubated with primary antibodies for 2 hours, and with secondary antibodies for one hour. 1∶1000 DAPI (Sigma: D9564) was added to the secondary antibody. Primary antibodies used: 1∶25 mouse anti-Zo-1 (Invitrogen: 33–9100), 1∶100 rabbit anti-aPkcζ/λ (Santa Cruz: sc-216), rabbit anti-β-catenin (CST: 9562), goat anti-Gata6 (R&D systems: AF1700), rabbit anti-Gata4 (Santa Cruz: Sc-9053), rabbit anti-Hnf4α (Santa Cruz: H-171), 1∶200 rat anti-Nanog (eBioscience: 14–5761), cleaved Caspase-3 (Cell signalling technology: 9661), 1∶250 rabbit anti-Fibronectin (Sigma: F3648). Secondary antibodies used: 1∶200 Alexa Fluor 488, goat Anti-rabbit IgG (Molecular Probes), 1∶500 Rhodamine Red-C-AffiniPure Donkey anti-rabbit IgG (Jackson ImmunoResearch), 1∶200 Dylight 550 goat Anti-mouse IgG (ImmunoReagents: GtxMu-003-E2550NHSX), 1∶500 AlexaFluor 488 goat anti-mouse (Molecular Probes), 1∶500 Fluoroscein Rabbit anti-goat IgG (Vector Laboratories), 1∶300 Alexa Fluor 488 goat Anti-rat IgG (Molecular Probes).

Embryoid bodies were washed five times with 0.1% tween in PBS following all antibody incubations. Stained embryoid bodies were then mounted in Mowiol (Calbiochem) within the middle of two 13 mm diameter stationery self-adhesive reinforcement rings stacked on top of each other, a coverslip was placed over the top. Immunostained embryoid bodies were examined on a Zeiss LSM510 Meta or a Leica SP5 laser-scanning confocal microscope. Image processing was done using Photoshop CS2 (Adobe) and Image J (NIH). Quantification was done by analysis of a field of view of at least three embryoid bodies per condition per experiment for at least three independent experiments. This gave at least 181 cells per experiment. Areas were randomly chosen using only the DAPI channel to avoid bias. Counts were done of surface layer cells only using the cell counter plugin in Image J (NIH). For statistical analysis a 1-way ANOVA with a Dunnetts post-hoc test, or a paired t-test were performed using GraphPad Prism 5.

### Biotinylation

The biotinylation method was adapted from previous protocols [28], [29]). Embryoid bodies were washed in PBS, and incubated with 10 mM EZ-Link Sulfo-NHS-LC-Biotin (Thermo Scientific 21335) diluted in DMEM for 30 minutes at room temperature. Two washes in DMEM were performed followed by a wash in PBS. Embryoid bodies were then fixed in 4% PFA in PBS for one hour, permeabilised in 0.25% triton (vol/vol) in PBS (Sigma), for 20 minutes and blocked for 30 minutes in 3% BSA (w/v) in 0.1% tween (vol/vol) in PBS. 1∶100 DyLight 488 conjugated Streptavidin (Thermo Scientific 21832) diluted in DMEM was added to embryoid bodies for 2 hours, they were subsequently washed five times with 0.1% tween in PBS. Embryoid bodies were mounted and imaged as stated in the immunofluorescence protocol.

## Results

### Embryoid Bodies Express Primitive Endoderm Cell Fate Markers by Day 7

**Figure 1:**
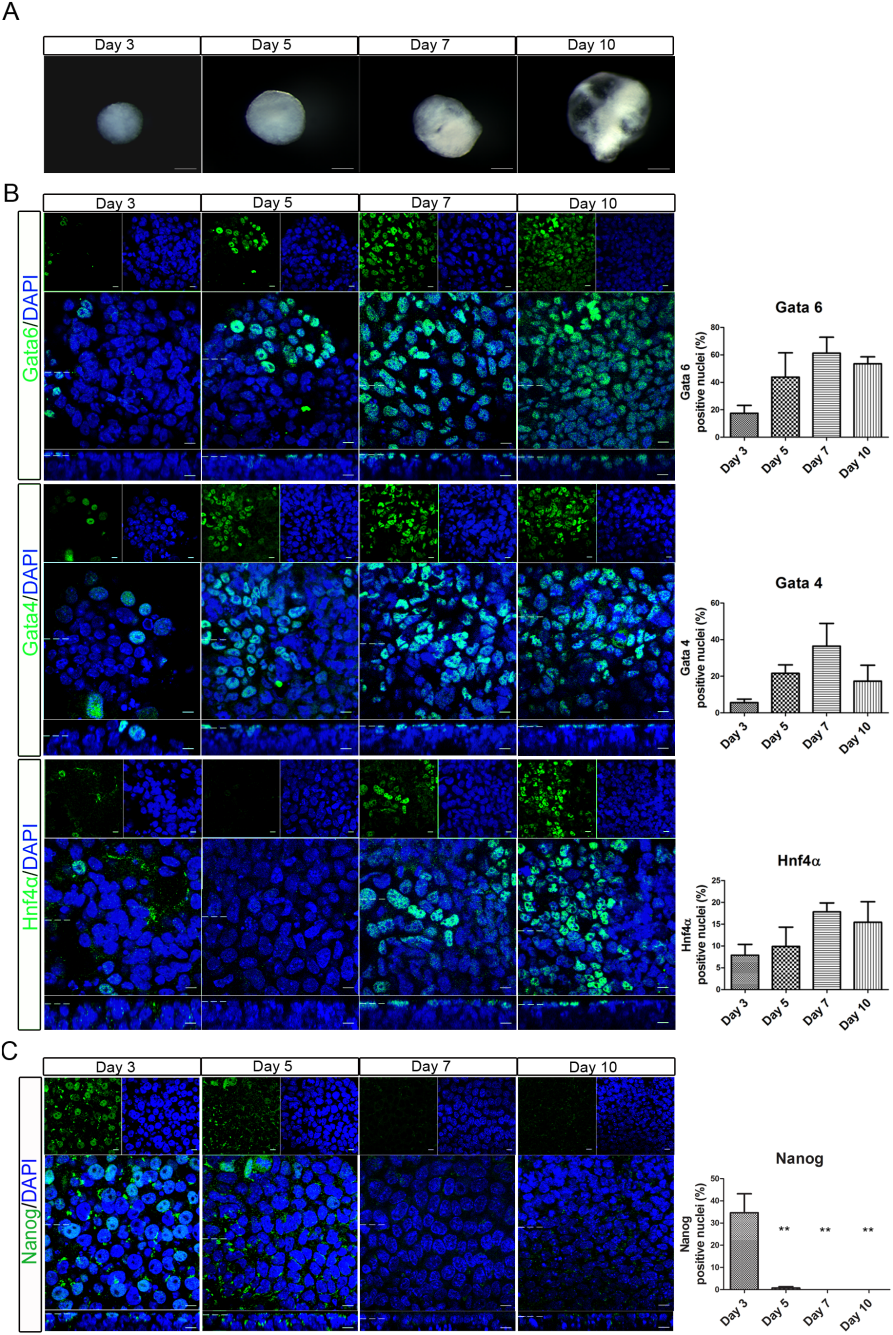
expression of primitive endoderm cell fate markers gradually increased and nanog decreased in developing embryoid bodies. Embryoid bodies were produced from R63 mES cells using the hanging drop method. Development of the embryoid body was monitored over ten days. (A) Light microscopy images show the gradual increase in size of the embryoid bodies as well as increased heterogeneity, loss of circularity and formation of cystic cavities at later timepoints. Scale bars 200 µm. (B) Whole-mount immunostaining showing nuclear localisation of Gata6, Gata4, and Hnf4α on days 3, 5, 7, and 10 of embryoid body development. The percentage of positive nuclei for each protein is shown graphically. The number of positive nuclei increased, reaching a maximum on day 7. (C) Whole-mount immunostaining showing nuclear localisation of Nanog. The number of positive nuclei rapidly decreased, with no positive cells seen on days 7 or 10. The percentage of positive nuclei for each protein is shown graphically. Data is from at least 3 independent experiments, error bars are standard error of the mean (SEM). Statistical analysis is a one-way Anova with a Tukey’s post-hoc test, (*P = 0.1–0.5, **p = 0.001–0.01, ***p<0.001). Dotted lines represent position that the relevant orthogonal or aerial images were taken. Scale bars 10 µm.

Before studying the polarity of the primitive endoderm in embryoid bodies, the temporal development of the primitive endoderm was characterised. A hanging-drop method was used to culture the embryoid bodies, and their morphology and expression of primitive endoderm transcription factor cell fate markers was examined. Embryoid bodies on day 3 were round and had a fairly homogeneous morphology (Figure 1A). Over time, the embryoid bodies showed increased heterogeneity and at day 7 and 10 balloon-like cysts were present (Figure 1A), which resembled those observed previously [30].

Whole-mount immunostaining of the outer-layer of the embryoid body showed that a small percentage of nuclei were positive for the primitive endoderm fate markers Gata6, Gata4 or Hnf4α on day 3 (Figure 1B). As the embryoid bodies grew, the number of nuclei which expressed these markers increased, reaching a peak on day 7 (Figure 1B). Additionally, expression of the pluripotency protein Nanog decreased (Figure 1C). It was expressed in approximately 35% of nuclei on day 3, this fell to 7% on day 5 and no cells expressed Nanog by day 7. Increasing expression of the primitive endoderm fate markers and loss of the pluripotency marker Nanog suggests that the outer layer of the embryoid bodies is undergoing a developmental program similar to that observed in the embryonic primitive endoderm.

### Primitive Endoderm of the Embryoid Body Develops Apico-basolateral Polarity by Day 5

**Figure 2:**
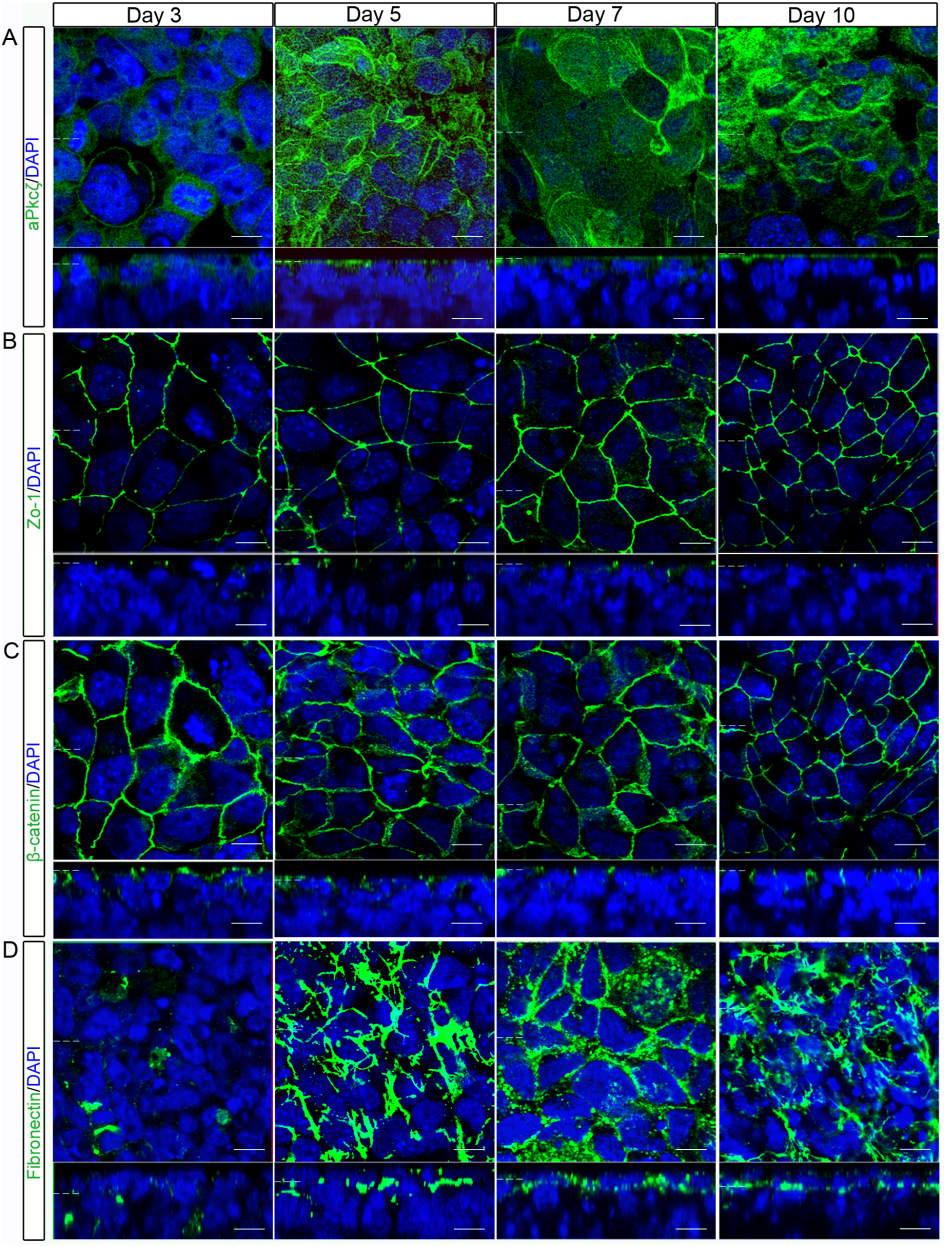
The outer-layer of embryoid bodies gradually developed apico-basolateral polarity. Localisation of proteins which show apico-basaolateral polarity in epithelia were examined in the outer, primitive endoderm layer of embryoid bodies using whole-mount immunostaining. (A) The polarity complex protein aPkcζ/λ showed cytoplasmic localisation on day 3, but was apically localised from day 5. (B) The tight-junction protein Zo-1 showed a polarised localisation from day 3 onwards. (C) The adherens-junction protein β-catenin showed apical and basolateral localisation at day 3, but by day 5 became more restricted to the lateral sides of cells. (D) The basement membrane protein Fibronectin formed aggregates on day 3, but from day 5 to day 10 showed gradually increasing staining at the basal side of the outer layer of cells. The outer cells remained polarised at 10 days. Representative images from 3 independent experiments are shown. Dotted lines represent position that the relevant orthogonal or aerial images were taken. Scale bars 10 µm.

The outer layer of the embryoid bodies appears to be following a developmental pathway which recapitulates primitive endoderm development. As polarity has been shown to change as the primitive endoderm forms [26], whole-mount immunostaining was used to observe the localisation of key markers of epithelial polarisation in embryoid bodies. After 3 days of culture, the embryoid bodies showed expression of the polarity complex protein aPkcζ/λ in the outer layer of cells, where it localised to the cytoplasm (Figure 2A). By day 5, aPkcζ/λ localisation was mostly apically restricted across the whole of the outer surface of cells of the embryoid body, this localisation remained unchanged on day 7 and 10. The tight-junction protein Zo-1 was localised to the border between the apical and lateral side of the epithelial cells, forming rings which outlined the cells by day 3 (Figure 2B). By day 5 the apical puncta were uniformly present in cells of the outer layer.

β-catenin, an adherens junction protein was predominantly present at the lateral side of cells of the outermost cell layer from day 3, but in some areas was also apically localised (Figure 2C). By day 5, β-catenin was consistently localised to the lateral side of the outer-most cell layer. Fibronectin is a basement membrane protein, on day 3 it localised to patches directly below the outermost cell layer, and deeper into the embryoid body (Figure 2D). This localisation gradually became more specific, by day 5 the staining was more restricted to the basal side of the outermost cell layer, whilst there were still patches of apical Fibronectin. By day 7 the Fibronectin was restricted to below the basal-side of the outermost cells and no staining was observed in cells below the outer cell layer. The gradual localisation of these apico-basolaterally polarised proteins shows that the epithelium surrounding the embryoid body has begun to polarise on day 3 and has clear polarisation by day 5.

### Inhibition of Mek with PD-0325901 Results in a Loss of Gata4 and Gata6 Expression in Embryoid Bodies

**Figure 3:**
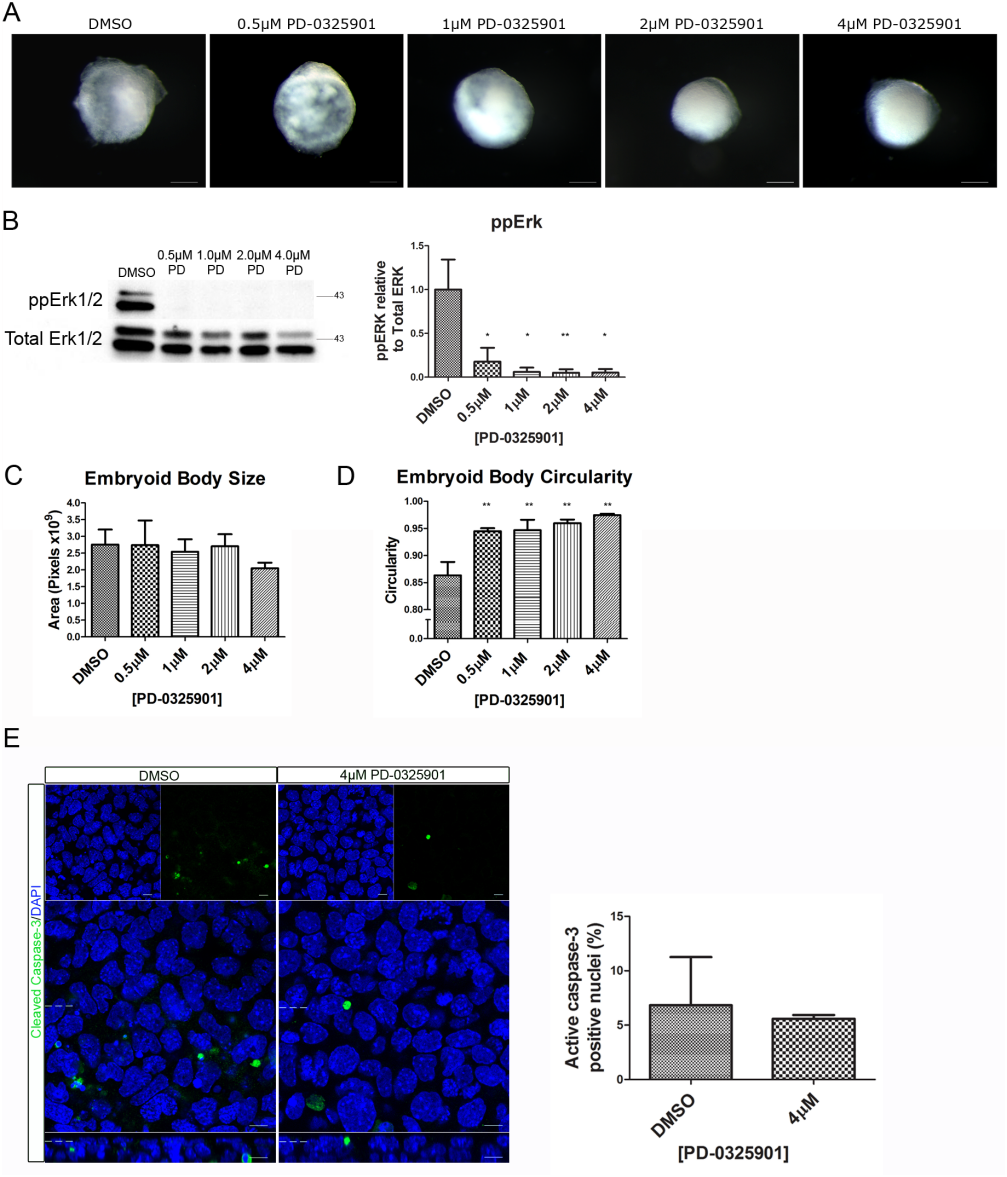
Addition of PD-0325901 inhibited Erk phosphorylation and resulted in more circular embryoid bodies. Embryoid bodies were grown in different concentrations of PD-0325901 or 0.04% DMSO for 7 days. (A) Light microscopy images show morphology of the embryoid bodies following inhibitor treatment. The embryoid bodies appeared slightly smaller and rounder. Scale bars 200 µm. (B) Western blotting demonstrates that PD-0325901 reduced levels of diphosphorylated Erk1/2. A representative blot and quantification from 3 independent experiments is shown. (C) Quantification of the size of the embryoid bodies suggests that there was a slight reduction in size, but this is not statistically significant. (D) Measurement of the circularity of the embryoid bodies shows that inhibition of Mek caused a statistically significant increase in circularity. (E) Whole-mount immunostaining of cleaved Caspase-3. A slight reduction in the number of cleaved Caspase-3 nuclei is observed upon treatment with 4 µM PD-0325901 in comparison to 0.04% DMSO. A representative image from 3 independent experiments is shown. Data is from 3 independent experiments, error bars are SEM. Statistical analysis is (B–D) a one-way Anova with a Dunnett’s post-hoc test, (E) a paired t-test. (*P = 0.1–0.5, **p = 0.001–0.01, ***p<0.001).

Erk signalling has previously been shown to have a role in primitive endoderm fate specification [10], [11], but its role in polarisation of these cells is less well established. To investigate the role of Mek signalling in this process PD-0325901, a potent Mek inhibitor [31], [32] was added to the cell suspension (day 0) and analysis of the phenotype of the embryoid bodies was carried out after 7 days of development (Figure 3A). Diphosphorylated Erk1 and 2 was markedly decreased (Figure 3B) demonstrating that, when added to the media PD-0325901 successfully inhibited Mek signalling. There was a slight reduction in size of the embryoid bodies at the highest dose tested (4 µM) in comparison to embryoid bodies treated with vehicle, but this was not statistically significant (Figure 3C). A statistically significant increase in the circularity of the embryoid body (Figure 3D) in comparison to control was observed. To establish if the addition of PD-0325901 caused an increase in apoptosis, whole-mount immunostaining for cleaved Caspase-3 was performed (Figure 3E). No significant change in the number of nuclei positive for cleaved Caspase-3 was observed in the outer layer of the embryoid bodies showing that inhibiting Mek did not cause a significant increase in apoptosis that might have affected the development of these cells.

**Figure 4:**
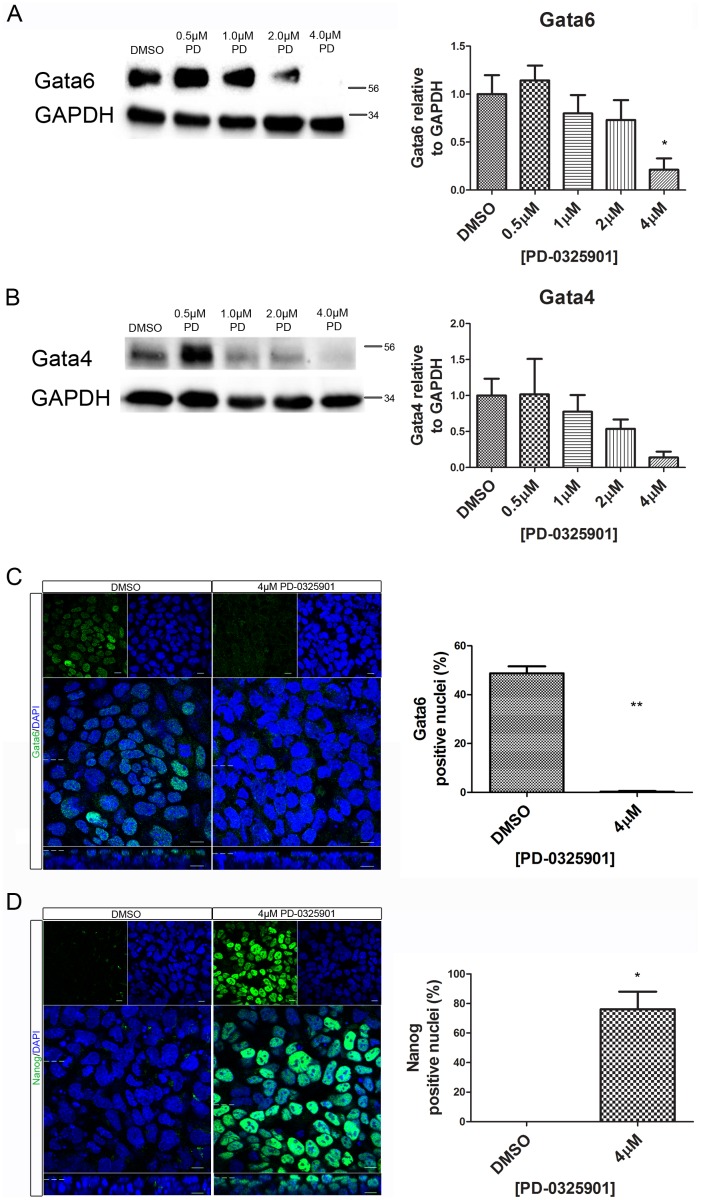
Reduced expression of primitive endoderm markers Gata4 and Gata6 and increased expression of Nanog was observed in embryoid bodies upon inhibition of Mek. Embryoid bodies were grown in different concentrations of PD-0325901 or 0.04% DMSO for 7 days. Expression levels of (A) Gata6, and (B) Gata4 were analysed using western blotting. A representative blot and quantification from 3 independent experiments is shown for each marker. A dose dependent decrease in expression of both proteins was observed. Statistical analysis is a one-way Anova with a Dunnett’s post-hoc test. Whole-mount immunostaining of (C) Gata6 and (D) Nanog after treatment of embryoid bodies with 4 µM PD-0325901. A reduction in the percentage of nuclei expressing Gata6 was observed whilst there was an increase in the percentage of nuclei expressing Nanog. A representative image from 3 independent experiments is shown. Scale bars 10 µm. Dotted lines represent position that the relevant orthogonal or aerial images were taken. Statistical test is a paired t-test. Data is from 3 independent experiments, error bars represent SEM. (*P = 0.1–0.5, **p = 0.001–0.01, ***p<0.001).

Disruption of Mek-signalling causes a loss of Gata6 and Gata4 expression, and an increase in Nanog expression preventing the fate specification of the primitive endoderm both *in vivo* and *in vitro* [6], [10]. The expression levels of Gata6, Gata4 and Nanog were analysed to assess the effect Mek inhibition has in this system. Western blotting of embryoid bodies showed a dose-dependent decrease in Gata6 and Gata4 protein expression levels, resulting in very little protein remaining at the highest concentration of PD-0325901 (4 uM) (Figure 4A & B). This result was confirmed using whole-mount immunostaining of Gata6 (Figure 4C). In contrast, the percentage of Nanog positive nuclei increased from 0% in control to an average of 76% upon addition of 4 µM PD-0325901 (Figure 4D). This confirms that upon inhibition of Mek the cells of the outer-layer of the embryoid body failed to become primitive endoderm cells, but instead remained pluripotent.

### Mek Inhibition Results in Disruption of Epithelial Polarity

**Figure 5:**
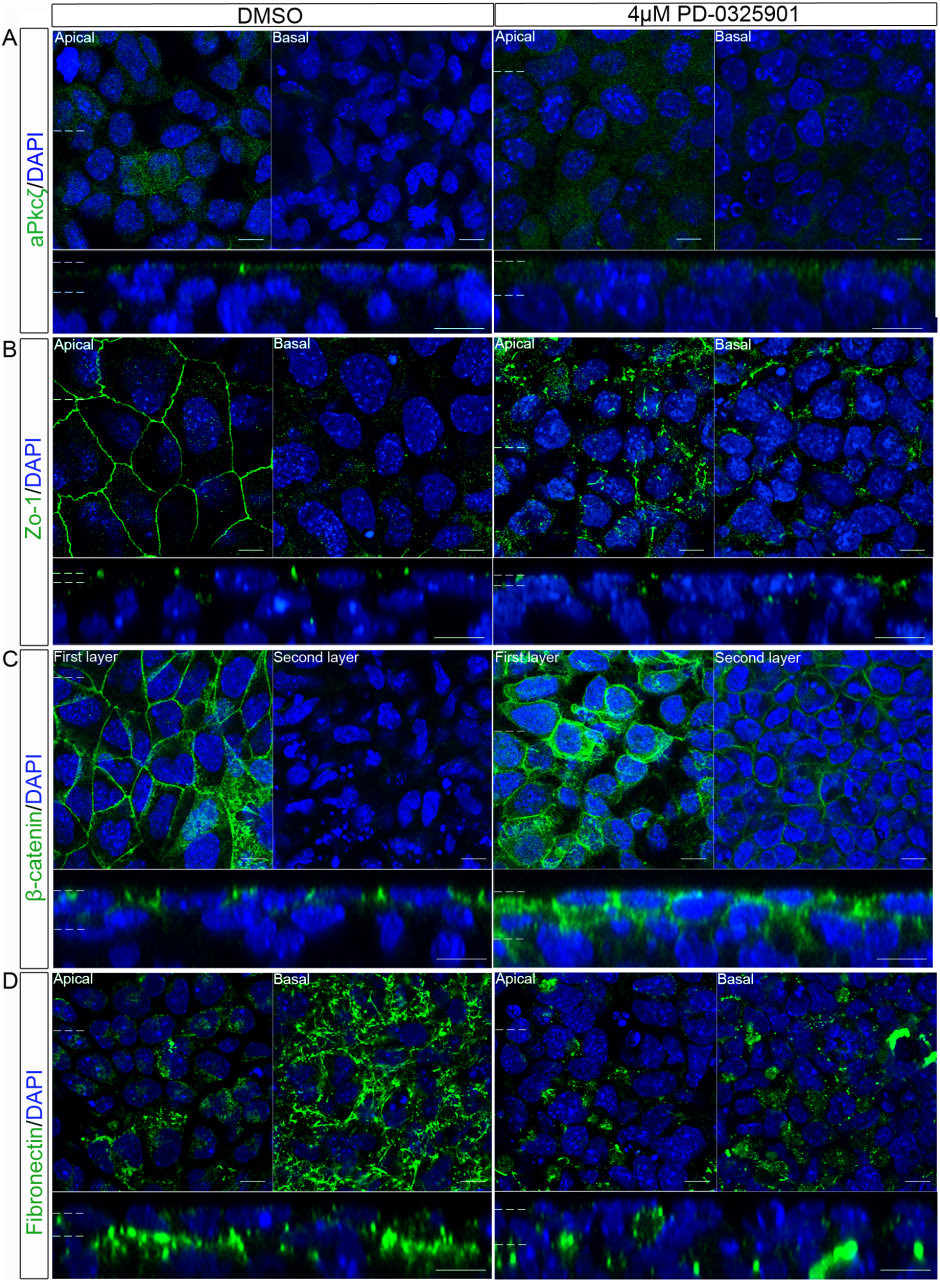
Inhibition of Mek disrupted the localisation of polarity and junction proteins in the outer layer of embryoid bodies. Embryoid bodies were treated with 4 µM PD-0325901 a Mek inhibitor, or 0.04% DMSO for 7 days. Localisation of proteins which normally polarise in the primitive endoderm epithelium were assessed using whole-mount immunostaining. (A) aPkcζ/λ a member of a polarity complex, (B) Zo-1 a tight junction protein, (C) β-catenin a protein in the adherens junction and (D) the basement membrane protein Fibronectin, were all shown to have an altered localisation when grown with a Mek inhibitor, suggesting a disruption in the apico-basolateral polarity of these cells. A representative image from 3 independent experiments is shown. Scale bars 10 µm. Dotted lines represent position that the relevant orthogonal or aerial images were taken.

Having observed a disruption in the specification of the primitive endoderm cell fate following inhibition of Mek signalling (Figure 2), embryoid bodies were cultured in PD-0325901 and the localisation of polarity and junctional proteins examined (Figure 2). The addition of 2 µM PD-0325901 appeared to cause maximal inhibition of ppErk1/2, but 4 µM caused a bigger reduction in Gata4/6 staining. The expression of these proteins in the primitive endoderm is Mek dependent [33] arguing that in this system there may be a low level of Mek signalling which is inhibited by addition of 4 µM PD-0325901. For this reason 4 µM PD-0325901 was chosen for subsequent experiments. aPkcζ/λ was apically localised in the control embryoid bodies, but when treated with the Mek inhibitor aPkcζ/λ was also present in the cytoplasm and in cell layers below the outer layer (Figure 5A). After treatment with the Mek inhibitor the tight-junction protein Zo-1 remained localised at the apical side of the outer layer cells but did not form a belt-like structure around the cells (Figure 5B). Occasional labelled junctions remained, but the majority of protein was present in isolated puncta. This suggested that inhibition of Mek disrupted the formation of the tight junctions and the localisation of aPkcζ/λ.

β-catenin localisation was also disrupted by treatment with the Mek inhibitor. It localised apically as well as laterally in the outer cell layer of embryoid bodies treated with PD-0325901 (Figure 5C) and was observed in the second layer of cells (the layer below the outer-layer). This localisation was not observed in the controls (Figure 5C, 2^nd^ layer panel). Lastly, a disruption in the localisation of the basement membrane protein Fibronectin was observed (Figure 5D). Upon addition of PD-0325901, Fibronectin formed small aggregates of protein instead of a fibrous network as observed in the control embryoid bodies. In addition, the Fibronectin protein in embryoid bodies treated with PD-0325901 was not basally restricted, but was present in all cell layers observed. The mislocalisation of aPkcζ/λ, as well as tight junction, adherens junction and basement membrane proteins shows that the apico-basolateral polarity of the outer layer of embryoid bodies was disrupted following Mek inhibition.

### Loss of Gata6 and Gata4 Expression upon Inhibition of Fgfr Signalling

**Figure 6:**
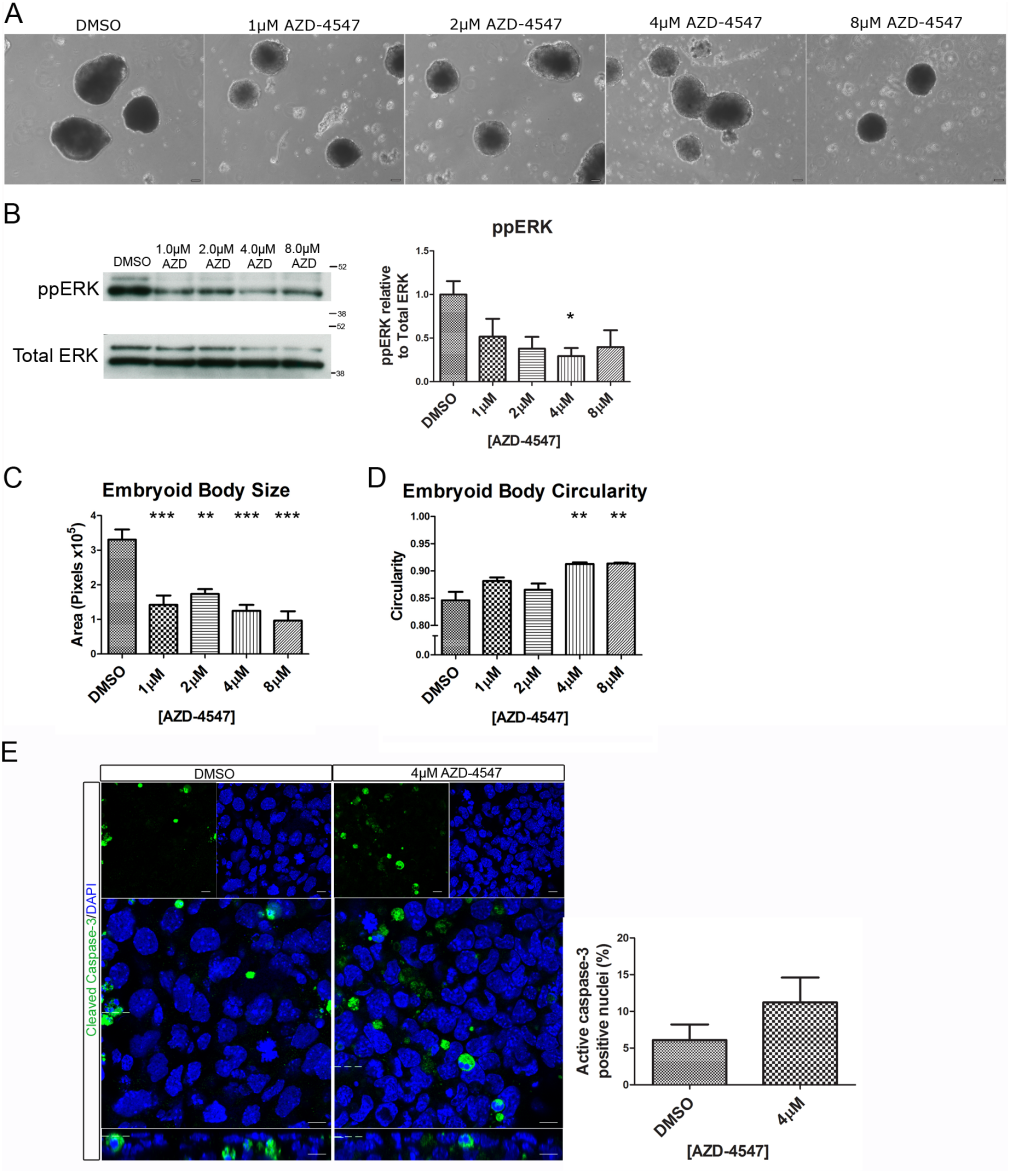
Addition of AZD-4547 inhibited Erk phosphorylation and resulted in smaller and more circular embryoid bodies. Embryoid bodies were grown in different concentrations of AZD-4547, 0.08% DMSO for 7 days. (A) Light microscopy images show a change in morphology of the embryoid bodies. Scale bars 100 µm. (B) Western blotting demonstrates that AZD-4547 reduced levels of diphosphorylated Erk1/2. A representative blot and quantification from 3 independent experiments is shown for each marker. (C) Inhibition of the Fgfr caused a significant reduction in size of the embryoid bodies. (D) Inhibition of the Fgfr caused a statistically significant increase in circularity. (E) Whole-mount immunostaining of cleaved Caspase-3 in embryoid bodies treated with 4 µM AZD-4547 or 0.04% DMSO. A small non-statistically significant increase in the number of cleaved Caspase-3 nuclei was observed upon treatment with AZD-4547 suggesting that more apoptosis may occur in the outer-layer of these embryoid bodies. A representative image from 3 independent experiments is shown. Data is from 3 independent experiments, error bars represent SEM. Statistical analysis is (B–D) a one-way Anova with a Dunnett’s post-hoc test, (E) a paired t-test (*P = 0.1–0.5, **p = 0.001–0.01, ***p<0.001).

As disruption of the Erk signalling cascade resulted in a disruption in the apico-basolateral polarity of the outer-layer of the embryoid body, we wished to identify the receptor responsible for Erk activation in this system. A leading candidate for this was the Fgfr, as its inhibition has previously been shown to decrease expression of Gata6 and Gata4, disrupting further fate specification of the primitive endoderm [13]–[15]. A newly developed potent and selective inhibitor of the Fgfr, AZD-4547, was used to treat embryoid bodies (Figure 6A). This inhibitor was chosen as it is more selective than previously available compounds such as PD-173074 [34], [35]. Addition of this inhibitor caused a reduction in ppERK1 and 2 (Figure 6B). Some ppErk expression remained, perhaps due to other signalling pathways upstream of Mek such as GPCRs, integrins or other receptor tyrosine kinases. Addition of AZD-4547 produced embryoid bodies which were smaller than controls even at the lowest dose tested (1uM) (Figure 6A&C). There was also a dose dependent increase in the circularity of the embryoid bodies, resulting in embryoid bodies which were significantly more circular than controls when cultured with 4 µM and 8 µM PD-0325901 (Figure 6A&D). To determine if addition of AZD-4547 caused an effect on apoptosis whole-mount immunostaining for cleaved Caspase-3 was carried out. There was a small, but not statistically significant, increase in the number of nuclei in the outer-layer of the embryoid body which were positive for cleaved Caspase-3 (Figure 6E). The majority of cells (>85%) remained Caspase negative, arguing that any changes in development of the primitive endoderm are not due to cell death.

**Figure 7:**
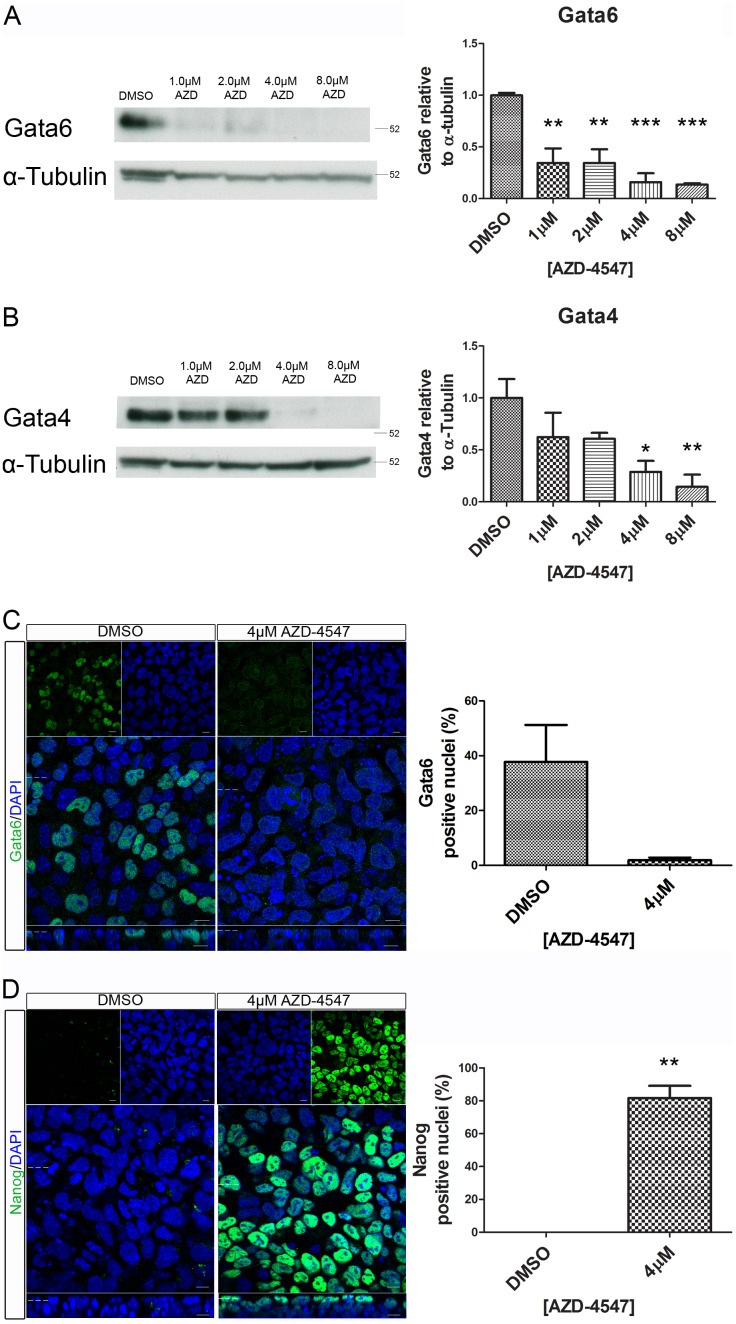
Reduced expression of the primitive endoderm markers Gata4 and Gata6 and increased expression of Nanog was observed in embryoid bodies following inhibitor of the Fgfr. Embryoid bodies were grown in different concentrations of AZD-4547 or 0.08% DMSO for 7 days. Expression levels of (A) Gata6, and (B) Gata4 were analysed using western blotting. A representative blot and quantification from 3 independent experiments is shown for each marker. A dose dependent decrease in expression of both proteins was observed. Statistical analysis is a one-way Anova with a Dunnett’s post-hoc test. Whole-mount immunostaining of (C) Gata6 and (D) Nanog after treatment of embryoid bodies with 4 µM AZD-4547 or 0.04% DMSO. A reduction in the percentage of nuclei expressing Gata6 was observed. The percentage of nuclei expressing Nanog increased. A representative image from 3 independent experiments is shown. Scale bars 10 µm. Dotted lines represent position that the relevant orthogonal or aerial images were taken. Statistical analysis is a paired t-test. Data is from 3 independent experiments, error bars represent SEM. (*P = 0.1–0.5, **p = 0.001–0.01, ***p<0.001).

A dose-dependent decrease in expression levels of both Gata4 and Gata6 was observed by western blotting when embryoid bodies were grown in increasing concentrations of the Fgfr inhibitor (Figure 7A&B). Additionally, whole-mount immunostaining confirmed a reduction in Gata6 positive nuclei when 4 µM AZD-4547 was used (Figure 7C). Nanog expression increased from 0% in control to an average of 82% upon inhibition of the Fgfr (Figure 7D). This suggests that Fgfr signalling is activating Erk to drive an increase in size, heterogeneity of shape, loss of Nanog, and expression of primitive endoderm fate markers in embryoid bodies.

### Loss of Apico-basolateral Polarity upon Fgfr Inhibition

**Figure 8:**
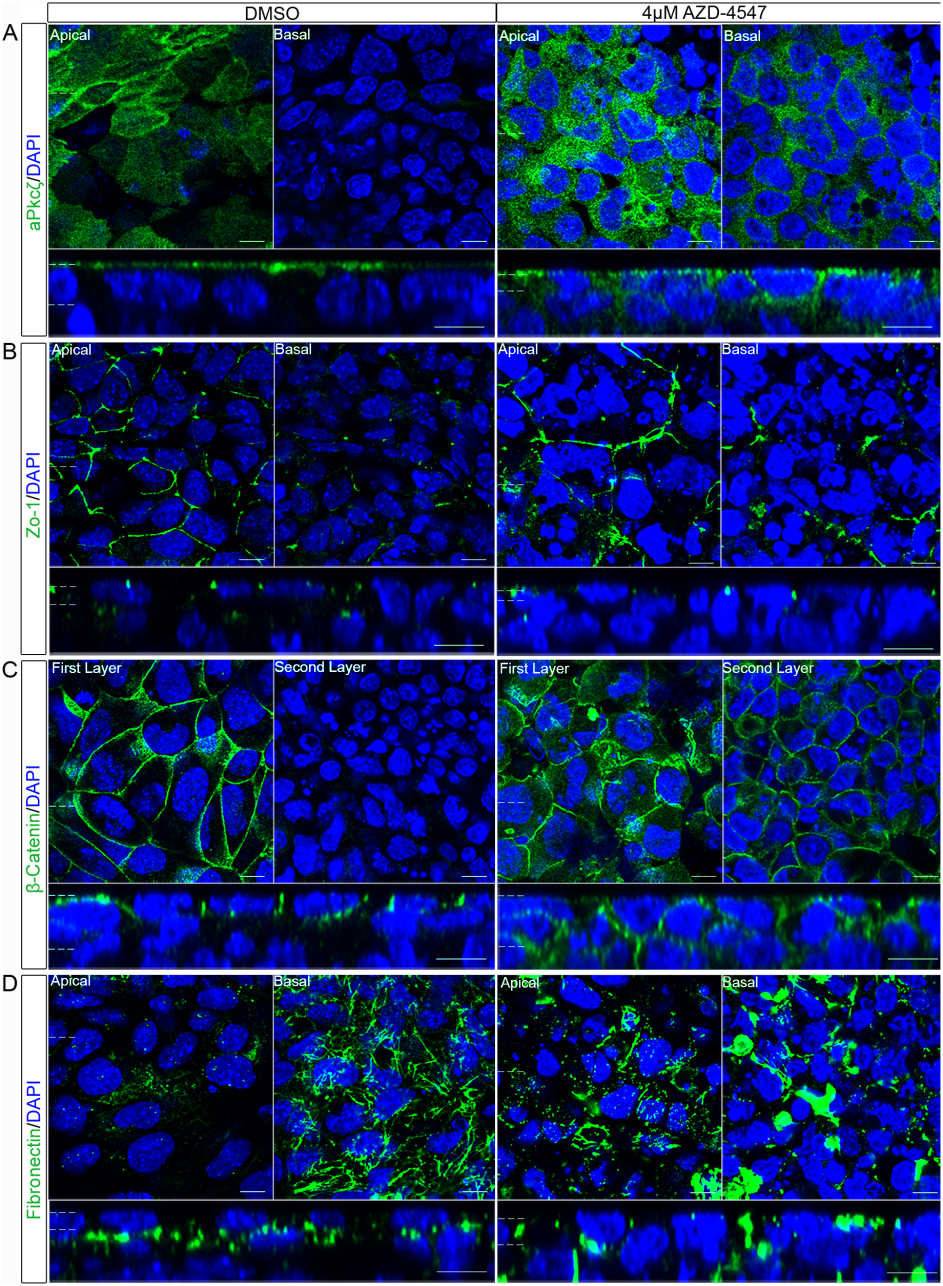
Inhibition of the Fgfr disrupted the normal localisation of polarity and junction proteins in the outer layer of embryoid bodies. Embryoid bodies were treated with the Fgfr inhibitor 4 µM AZD-4547, or 0.04% DMSO for 7 days. Whole-mount immunostaining demonstrated the localisation of proteins normally polarised in the primitive endoderm. (A) aPkcζ/λ a member of a polarity complex, (B) Zo-1 a tight junction protein, (C) β-catenin a protein in the adherens junction and (D) the basement membrane protein Fibronectin, were shown to lose their apico-basolateral polarised localisation when embryoid bodies were grown with an Fgfr inhibitor suggesting a disruption in the apico-basolateral polarity of these cells. A representative image from 3 independent experiments is shown. Scale bars 10 µm. Dotted lines represent position that the relevant orthogonal or aerial images were taken.

Having observed a loss in expression of primitive endoderm markers, we examined apico-basolateral polarity components following inhibition of the Fgfr. This analysis was performed using 4 µM AZD-4547 as a clear inhibition of ppERK, loss of Gata6 and Gata4 expression and, morphological changes were observed at this concentration. Following inhibition of the Fgfr, aPkcζ/λ localised throughout the cytoplasm of the outer-layer and was found in the layers below (Figure 8A). A disruption in both adherens junctions and tight junction proteins was also observed. Zo-1 localised to apical puncta, but did not form a belt around the periphery of the cells, instead only patches or short lines of Zo-1 were detected (Figure 8B). β-catenin was observed apically in some cells and was also found in the cells below the outer layer (Figure 8C). Localisation of Fibronectin was also investigated. The protein was spread throughout the outer layer, as well as in layers below and was present in patches, rather than forming the fibrous network observed in controls (Figure 8D). The mislocalisation of these proteins was similar to that seen when embryoid bodies were treated with the Mek inhibitor, suggesting that Mek dependent polarisation is driven by activation of the Fgfr.

### Inhibition of Mek Or Fgfr Signalling does not Significantly Alter the Expression Levels of Polarity and Junction Proteins

**Figure 9:**
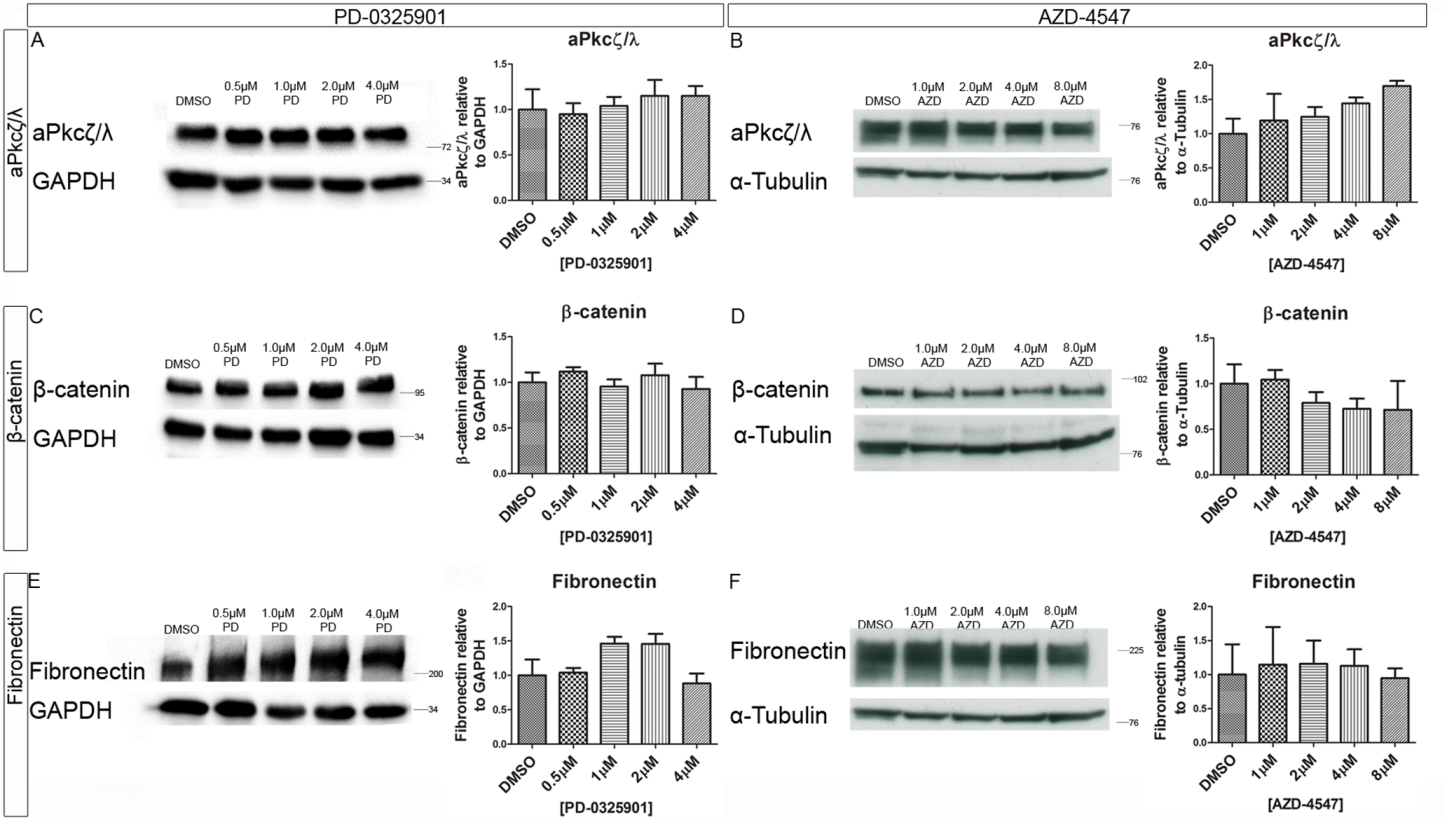
Inhibition Mek or the Fgfr did not significantly alter the expression levels of polarity and junction proteins. Embryoid bodies were grown in different concentration of the (A, C, E), Mek inhibitor PD-0325901 and 0.04% DMSO or the (B, D, F) Fgfr inhibitor AZD-4547 and 0.08% DMSO for 7 days. Expression levels of polarity and junction proteins were assessed using western blotting (A&B) aPkcζ/λ, (C&D) β-catenin, and (E&F) Fibronectin. A representative blot and quantification from 3 independent experiments is shown for each marker. No statistically significant change was seen in any of the markers observed. Statistical analysis is a one-way Anova with a Dunnett’s post-hoc test. Error bars represent SEM. (*P = 0.1–0.5, **p = 0.001–0.01, ***p<0.001).

To determine whether inhibition of Mek or the Fgfr altered the levels of the polarity proteins, the expression of aPkcζ/λ, β-catenin, Fibronectin were assessed following treatment with PD-0325901 or AZD-4547 using western blotting (Figure 9). Quantification suggested some small changes following inhibitor treatment, but none of these were statistically significant. This argues that, although small changes cannot be ruled out, inhibition of Mek or the Fgfr predominantly caused a re-localisation of these proteins rather than a change in their expression levels.

### Inhibition of the Fgfr with PD-173074 Causes a Disruption in Polarisation of the Outer-layer of Cells

Treatment of embryoid bodies with the recently developed Fgfr inhibitor AZD-4547 caused a mis-localisation of apico-basolaterally polarised proteins. To confirm the specificity of this effect and allow comparison with previous literature, the polarisation of embryoid bodies was observed using the structurally distinct and more commonly used inhibitor PD-173074. This is an effective inhibitor of Fgfr1, but also inhibits the Vascular endothelial growth factor receptor 2 (Vegfr2) [35]. The polarisation of the primitive endoderm was observed following treatment with 0.1 µM PD-173074 which is the concentration commonly used for studying primitive endoderm *in vivo* [16], [17]. In these *in vivo* studies it is often used in combination with a Mek inhibitor [16], [17]. Embryoid bodies were therefore also treated with 0.1 µM PD-173074 and 1 µM PD-0325901, the combination and concentration of inhibitors previously used to assess primitive endoderm development [16], [17].

**Figure 10:**
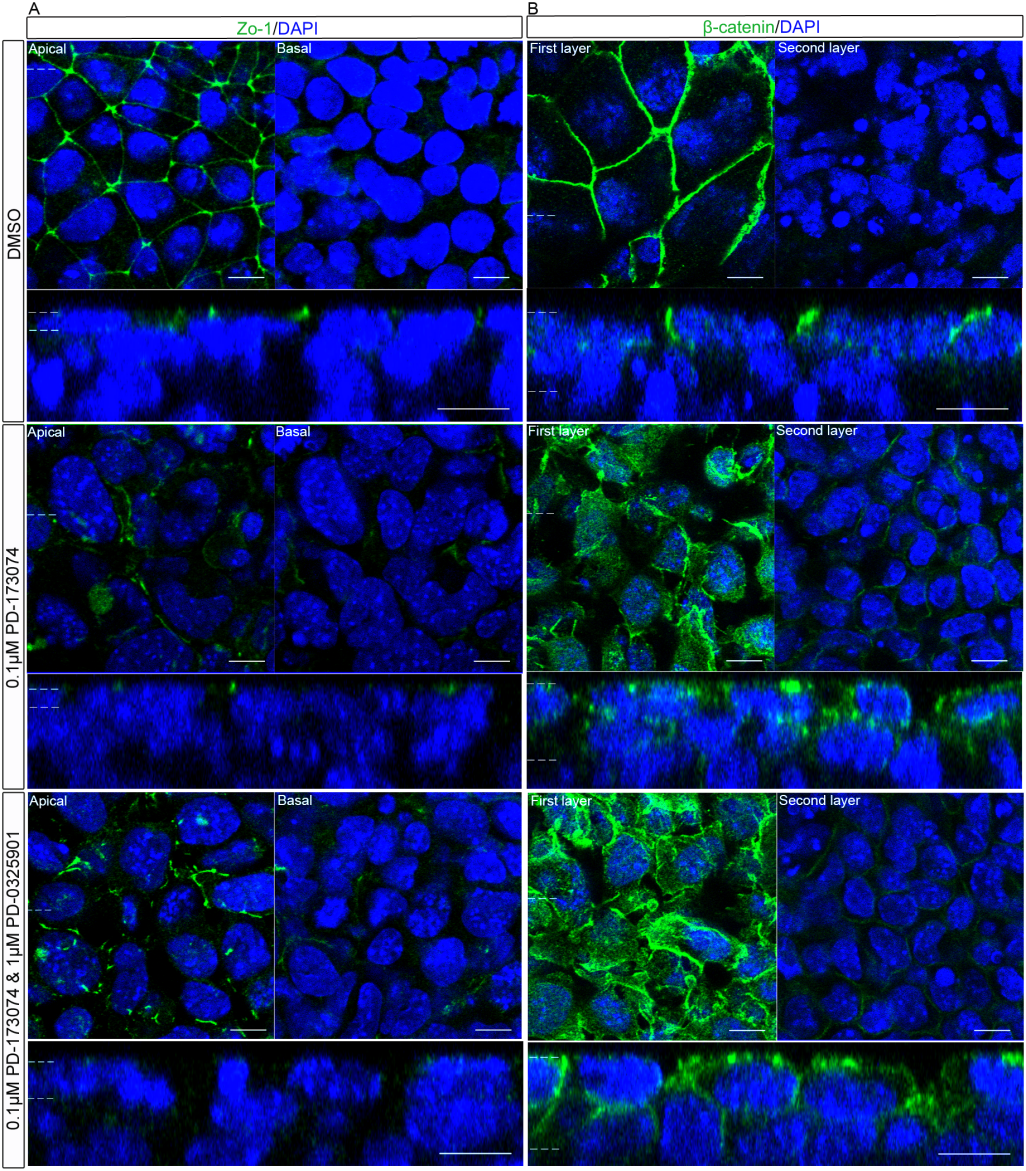
Inhibition of the Fgfr with PD-173074 or the Fgfr and Mek disrupted the normal localisation of polarity and junction proteins in the outer layer of embryoid bodies. Embryoid bodies were treated with 0.1 µM of the Fgfr inhibitor PD-173074, 0.1 µM PD-173074 and 1 µMPD-0325901 or 0.02% DMSO for 7 days. Whole-mount immunostaining demonstrated the localisation of proteins normally polarised in the primitive endoderm. A) Zo-1 a tight junction protein, and (B) β-catenin a protein in the adherens junction were shown to lose their normal polarised localisation when embryoid bodies were incubated with 0.1 µM PD-173074 or 0.1 µM PD-173074 and 1 µM PD-0325901. This suggests a disruption in the apico-basolateral polarisation of these cells. A representative image from 3 independent experiments is shown. Scale bars 10 µm. Dotted lines represent position that the relevant orthogonal or aerial images were taken.

The tight-junction protein Zo-1 localised to junctions in control embryoid bodies, but this localisation was disrupted following treatment with either 0.1 µM PD-173074 or the combination of 0.1 µM PD-173074 and 1 µM PD-0325901 (Figure 10A). The localisation of the adherens junction protein β-catenin was also disrupted following treatment with PD-173074 and when treated with the combined inhibitors (Figure 10B). The use of two structurally distinct inhibitors of the Fgfr, which gave very similar results, argues that the phenotypes described above are caused by specifically inhibiting Fgfr signalling.

### Inhibition of Mek or the Fgfr Signalling Results in a Loss of Epithelial Barrier Function

**Figure 11:**
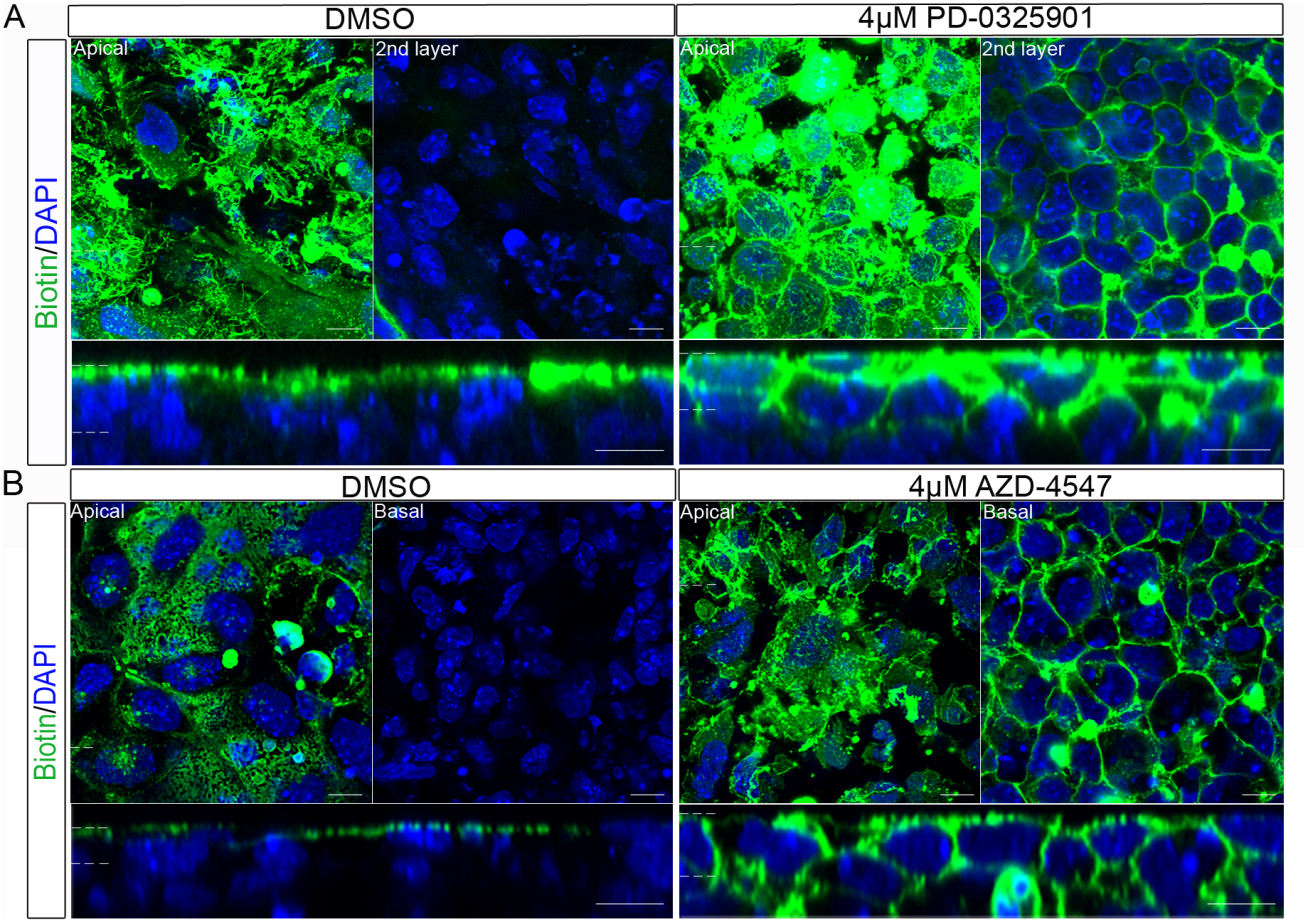
Inhibition of the Fgfr or Mek caused a loss in barrier function of the cells in the outer layer of the embryoid body. Embryoid bodies were treated with (A) 0.04% DMSO, or 4 µM PD-0325901(Mek inhibitor), (B) 0.04% DMSO or 4 µM AZD-4547 (Fgfr inhibitor) for 7 days. A membrane impermeable biotin which covalently links to amino groups was added to the embryoid bodies and subsequently visualised using Alexa-fluor-488-conjugated Streptavidin. The biotin was largely restricted to the apical surface of the embryoid body in DMSO controls. Following treatment with either a Mek or an Fgfr inhibitor, biotin also bound to basolateral membrane proteins of the outer cell layer and the membranes of cells under this layer. This shows that the normal epithelial barrier has been disrupted. A representative image from 3 independent experiments is shown. Scale bars 10 µm. Dotted lines represent position that the relevant orthogonal or aerial images were taken.

The maintenance of a diffusion barrier is a common function of epithelial layers [36]. We therefore investigated if, in addition to the mislocalisation of apico-basolateral polarity proteins described above, the cells of the embryoid bodies had defects in their barrier function. The diffusion of a membrane-impermeable biotin, which covalently links to amino groups on extracellular proteins, was used as a test for epithelial barrier function. The bound biotin can be readily visualised using fluorescently labelled streptavidin. In control embryoid bodies (Figure 11), binding of biotin was restricted to the outer, apical layer of the embryoid bodies. When embryoid bodies were treated with the Mek inhibitor (PD-0325901) or Fgfr inhibitor (AZD-4547), the biotin was observed throughout the lateral side of the outer-layer cells, and in deeper cell layers (Figure 11). This demonstrated that the barrier function of the embryoid body epithelium was disrupted following inhibition of signalling by Mek or the Fgfr.

## Discussion

### Summary

Here, we investigate polarisation of the primitive endoderm using embryoid bodies as a model system. Our data demonstrates a gradual increase in expression of primitive endoderm markers, loss of Nanog expression and establishment of epithelial polarisation in the primitive endoderm layer of embryoid bodies. Inhibition of both Mek and the Fgfr caused a loss of primitive endoderm markers, maintenance of Nanog and mislocalisation of all the polarity proteins examined; polarity complex protein aPkcζ/λ, tight junction protein Zo-1, adherens junction protein β-catenin, and basement membrane protein Fibronectin. Associated with the disruption in polarity and junctional protein localisation was a loss of the barrier function of the epithelium. This argues that Fgfr signalling activates Erk 1 and 2 which then promotes cell fate specification, polarisation and establishment of a functional epithelial barrier.

### Embryoid Bodies as a Model of Epithelial Polarity

The polarised localisation of a number of proteins has been previously reported in the primitive endoderm [20]–[24], however, the temporal localisation of multiple epithelial polarity proteins has not previously been examined. The results presented here show that the primitive endoderm of an embryoid body gradually polarises in an order which is similar to that observed in other epithelia (Reviewed by [37]). Zo-1 and β-catenin localise at junctions at the initial stage of cell polarisation, followed by localisation of aPkcζ/λ at the apical membrane and deposition of Fibronectin to form a basement membrane.

There are many different models which can be used to study epithelial polarisation, including model organisms such as *Drosophila melanogaster*, polarised cell culture cells such as MDCK cells, and epithelial organs from embryonic and adult mice [38]–[40]. However, the primitive endoderm of an embryoid body has a number of characteristics which we believe will make it a valuable model for future experiments. It is a 3 dimensional model composed of a variety of non-transformed cell types, which gradually polarise and express cell fate markers. There are also a growing number of knockout and transgenic ES cell lines which could be studied in this system [41], [42]. In particular, embryoid bodies are well suited for studying the relationship between polarisation and fate specification in a developing epithelium.

### Fgfr/Erk Signalling is Required for Cell Fate Specification and Polarisation of the Primitive Endoderm

The Fgfr/Erk signalling pathway is known to be required for cell fate specification of the primitive endoderm [9]. Our data confirms that inhibition of either the Fgfr or Mek signalling causes a loss of primitive endoderm cell fate markers and maintenance of the pluripotency protein Nanog. Interestingly, inhibition of the Fgfr caused a stronger reduction of primitive endoderm fate markers, despite inducing less inhibition of Erk than the Mek inhibitor. This suggests that another receptor may be activating Mek. It also raises the possibility that the Fgfr might be activating other signalling pathways, such as Pi3k-Akt, Stat, Plcγ, p38 MapK, and Jnk [43] to promote expression of Gata4 and Gata6. These additional pathways may regulate embryoid body size, as the Fgfr inhibitor caused a bigger reduction in size than addition of the Mek inhibitor. Another interesting phenotype seen following inhibition of both Fgfr or Mek was an increase in circularity. This may be caused by a failure to become cystic, causing the embryoid bodies to remain more homogeneous. Gata4^−/−^ embryoid bodies are also smaller than controls and non-cystic [44] and embryoid bodies made with Laminin γ1^−/−^ mES cells do not cavitate [45]. Therefore, the increase in circularity may be linked to a failure in primitive endoderm development and polarisation.

Our data also show that Fgfr/Erk signalling is required for the formation of a polarised primitive endoderm layer with an efficient barrier function. This hypothesis is supported by a several other findings. Firstly, aPkc does not polarise in the primitive endoderm of embryos grown in 2i medium, which includes a Mek and a GSK3β inhibitor [26]. Secondly, expression of a truncated form of Fgfr2 in mES cells has been reported to disrupt basement membrane formation [46], while Grb2 knockout embryoid bodies show an absence of Laminin basal to the outer cell layer [10]. Combining our data with previous studies leads us to propose that the Fgfr/MAPK signalling pathway promotes polarisation of the primitive endoderm as well as its fate. There are at least three possibilities to explain this dual role; 1) Fgfr/Erk signalling regulates primitive endoderm cell fate specification which then promotes polarisation. 2) Fgfr/Erk signalling initiates the polarisation of the primitive endoderm which then drives fate specification. 3) MAPK independently regulates both the polarisation and fate determination of the primitive endoderm.

In embryoid bodies, the cells start to polarise prior to the maximal nuclear localisation of Gata6, Gata4, and Hnf4α (Figures 1 and 2). This suggests that in embryoid bodies the onset of polarisation occurs before cell fate specification and argues against model one where cell fate regulators promote polarisation. There is also evidence that disrupting polarisation of the primitive endoderm does not affect fate determination, arguing against model two. Embryoid bodies formed with mES cells which do not express Laminin γ1, a key component of the basement membrane, have primitive endoderm cells in the outer-layer although they were not properly organised [24], [45]. Additionally, E-cadherin ^−/−^ ES cells showed normal primitive endoderm development, but the embryoid bodies did not form a cavity, consistent with defects in epithelial barrier formation [23]. In Dab2 null mouse embryos cells of the primitive endoderm lose their apico-basolateral polarity. In these embryos Gata4 expression was observed, but positive cells were positioned throughout the epiblast, suggesting a failure in positioning but not fate regulation [22], [23].

The studies described above argue against the idea of polarity regulating cell fate (model 2). However, there is also conflicting evidence that loss of proteins required for epithelial polarisation can disrupt primitive endoderm fate specification. In Integrin β1−/− embryoid bodies, α-fetoprotein (a marker of visceral endoderm) is only expressed in clusters of endoderm cells, Dab2 protein was not observed by immunostaining, and Gata4 became cytoplasmic [47]. This suggests that the absence of integrins disrupts the development of the primitive endoderm. The mutant embryoid bodies had no diphospho-Erk1/2, and had reduced p38 and Akt activation suggesting that Integrins control endoderm differentiation via the Raf/Erk and Akt signalling pathways [47]. Live-imaging of embryos treated with an aPkc inhibitor revealed that the primitive endoderm cells migrate but fail to remain on the ICM surface when they reach the cavity. This resulted in primitive endoderm cells being present throughout the epiblast rather than forming a distinct layer on the edge of the blastocoel [26]. No effect was seen on early markers of the primitive endoderm fate (Pdgfra, and Gata6), but some cells were negative for both Nanog and Gata4, suggesting a defect in primitive endoderm maturation [26]. Co-transfection experiments also showed that the Dab2 promoter can be transactivated by forced expression of Gata6, in NIH-3T3 cells [48] suggesting the polarity regulator Dab2 may be a downstream target of the cell fate regulator Gata6. In summary, current evidence appears to support the idea of a hybrid model where early regulation of primitive endoderm fate acts upstream of polarisation, but that aPkc and possibly other regulators of polarity, are required for the maturation of the primitive endoderm fate. This is clearly a complex process, and the mechanistic elucidation of Fgfr/Erk signalling’s dual role in cell fate determination and cell polarisation, and how these two processes are interlinked and interact will be a key aim goal for future work.

### Conclusion

In conclusion, our results demonstrate that the Fgfr/Erk pathway is required for the formation of a polarised primitive endoderm cell layer in embryoid bodies. Further studies into the mechanisms responsible for this requirement and the relationship between cell fate specification and cell polarity are needed to improve our understanding of the development of this key embryonic cell type. These studies are likely to be relevant to the development of other embryonic and adult epithelia.
