# Correction: Formation of a Polarised Primitive Endoderm Layer in Embryoid Bodies Requires Fgfr/Erk Signalling

**DOI:** 10.1371/journal.pone.0141401

**Published:** 2015-10-30

**Authors:** Gail Doughton, Jun Wei, Nicolas Tapon, Melanie J. Welham, Andrew D. Chalmers

The authors would like to correct Figs 6, 7 and 9, as well as the caption for Fig 2.

In Fig 2, the images for rows B and C for conditions Day 3, Day 7, and Day 10 were carried out as co-immunofluorescence, so the DAPI stain is the same for both markers. The authors have provided a corrected caption for Fig 2 to clarify this.

**Fig 2:**
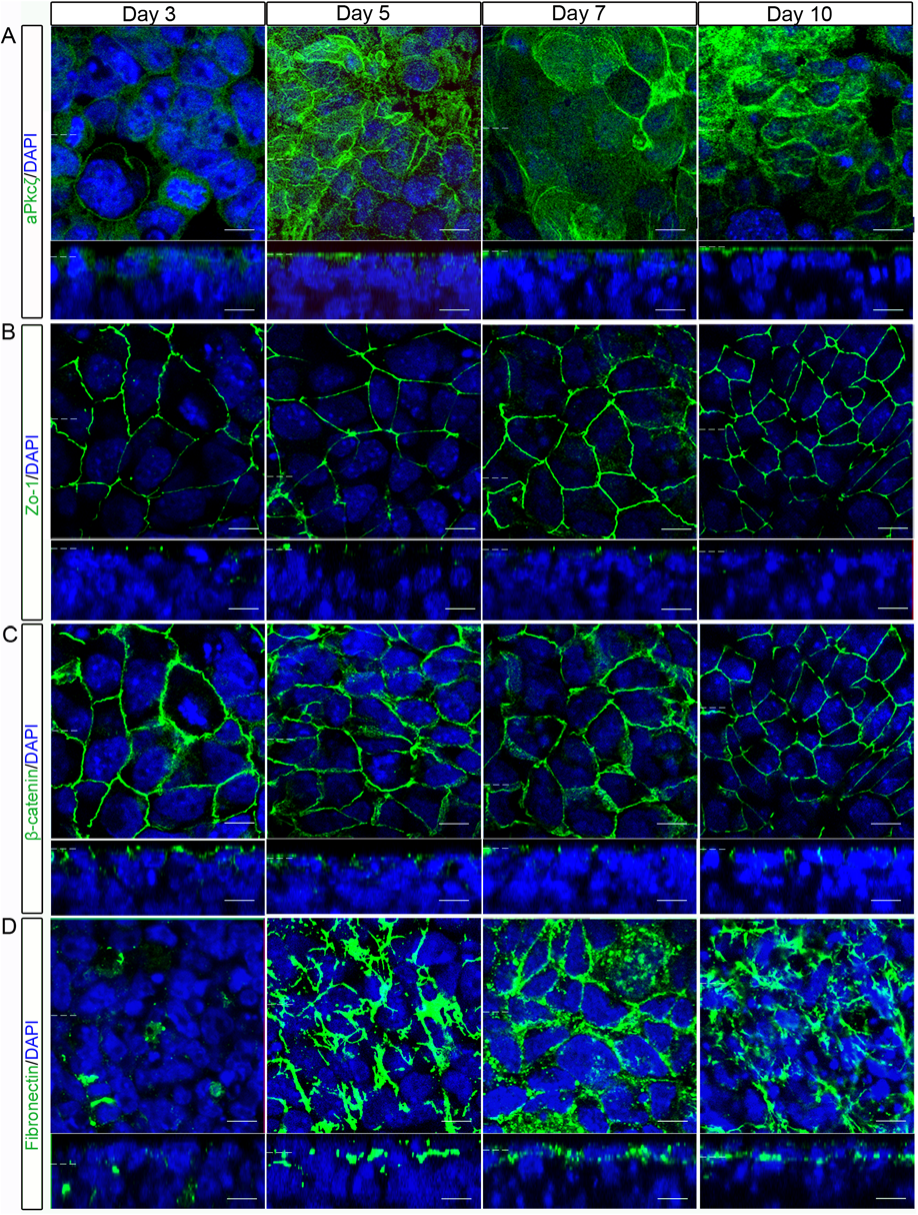
The outer-layer of embryoid bodies gradually developed apico-basolateral polarity. Localisation of proteins which show apico-basaolateral polarity in epithelia were examined in the outer, primitive endoderm layer of embryoid bodies using whole-mount immunostaining. (A) The polarity complex protein aPKCζ/λ shows cytoplasmic localisation on day 3, but was apically localised from day 5. (B)The tight-junction protein Zo-1 showed a polarised localisation from day 3 onwards. (C) The adherens-junction protein β-catenin showed apical and basolateral localisation at day 3, but by day 5 became more restricted to the lateral sides of cells. The Zo-1 and β-catenin staining (B and C) for days 3, 7 and 10 were carried out as co-immunofluorescence, so the DAPI stain is the same for both markers. The Zo-1 staining was originally red, but is shown as green for consistency with other images. (D) The basement membrane protein Fibronectin formed aggregates on day 3, but from day 5 to day 10 showed gradually increasing staining at the basal side of the outer layer of cells. The epithelia remained polarised at 10 days. Representative images from 3 independent experiments are shown. Dotted lines represent position that the relevant orthogonal or aerial images were taken. Scale bars 10μm.

In Fig 6, two of the images (1 µM AZD-4547 and 2 µM AZD-4547) were mistakenly derived from the same sample. The authors have provided a revised version of Fig 6 with a corrected image for 1 µM AZD-4547.

**Fig 6:**
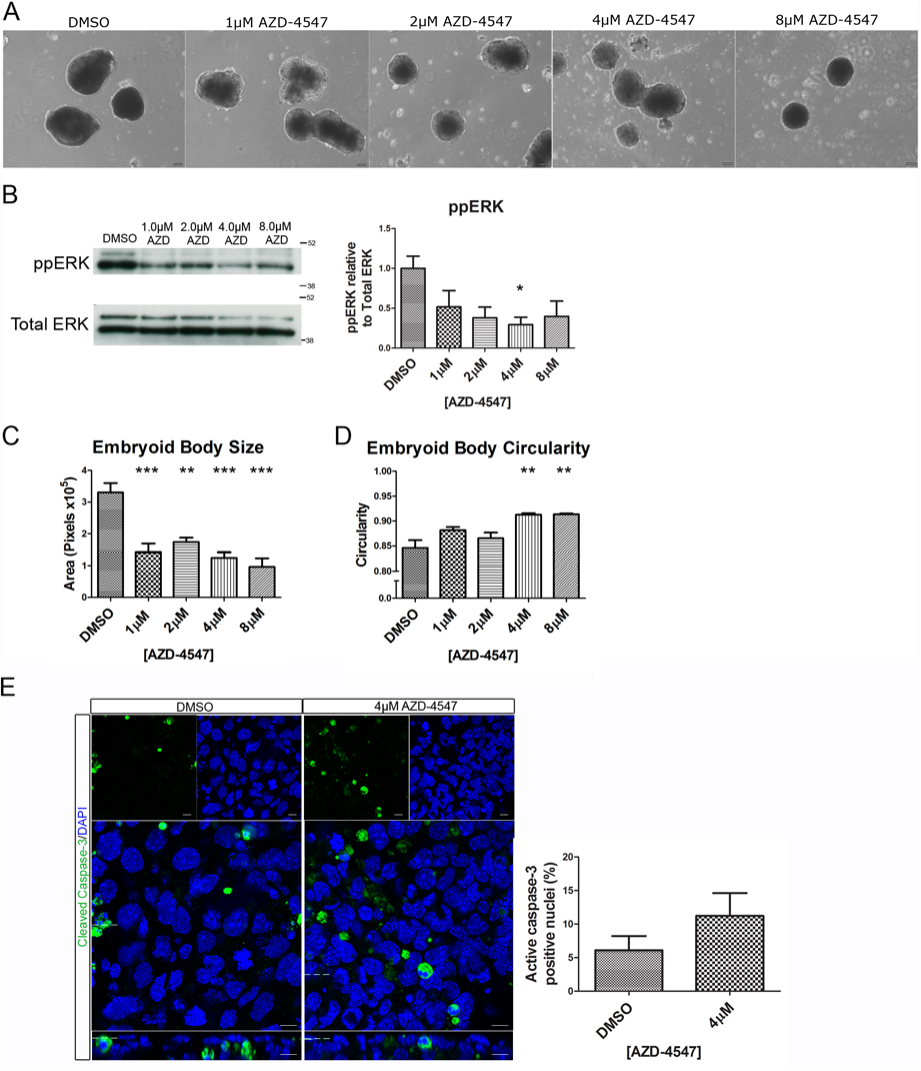
Addition of AZD-4547 inhibited Erk phosphorylation and resulted in smaller and more circular embryoid bodies. Embryoid bodies were grown in different concentrations of AZD-4547, 0.08% DMSO for 7 days. (A) Light microscopy images show a change in morphology of the embryoid bodies. Scale bars 100μm. (B) Western blotting demonstrates that AZD-4547 reduced levels of diphosphorylated Erk1/2. A representative blot and quantification from 3 independent experiments is shown for each marker. (C) Inhibition of the Fgfr caused a significant reduction in size of the embryoid bodies. (D) Inhibition of the Fgfr caused a statistically significant increase in circularity. (E) Whole-mount immunostaining of cleaved Caspase-3 in embryoid bodies treated with 4μM AZD-4547 or 0.04% DMSO. A small non-statistically significant increase in the number of cleaved Caspase-3 nuclei was observed upon treatment with AZD-4547 suggesting that more apoptosis may occur in the outer-layer of these embryoid bodies. A representative image from 3 independent experiments is shown. Data is from 3 independent experiments, error bars represent SEM. Statistical analysis is (B-D) a one-way Anova with a Dunnett’s post-hoc test, (E) a paired t-test (* P = 0.1–0.5, ** p = 0.001–0.01, *** p<0.001).

The stains in Fig 7D (right panel) are an incorrect duplicate of Fig 4D (right panel). The authors have provided a corrected Fig 7 here. The left panels of Fig 7D are correctly duplicated as they are both identical DMSO controls.

**Fig 7:**
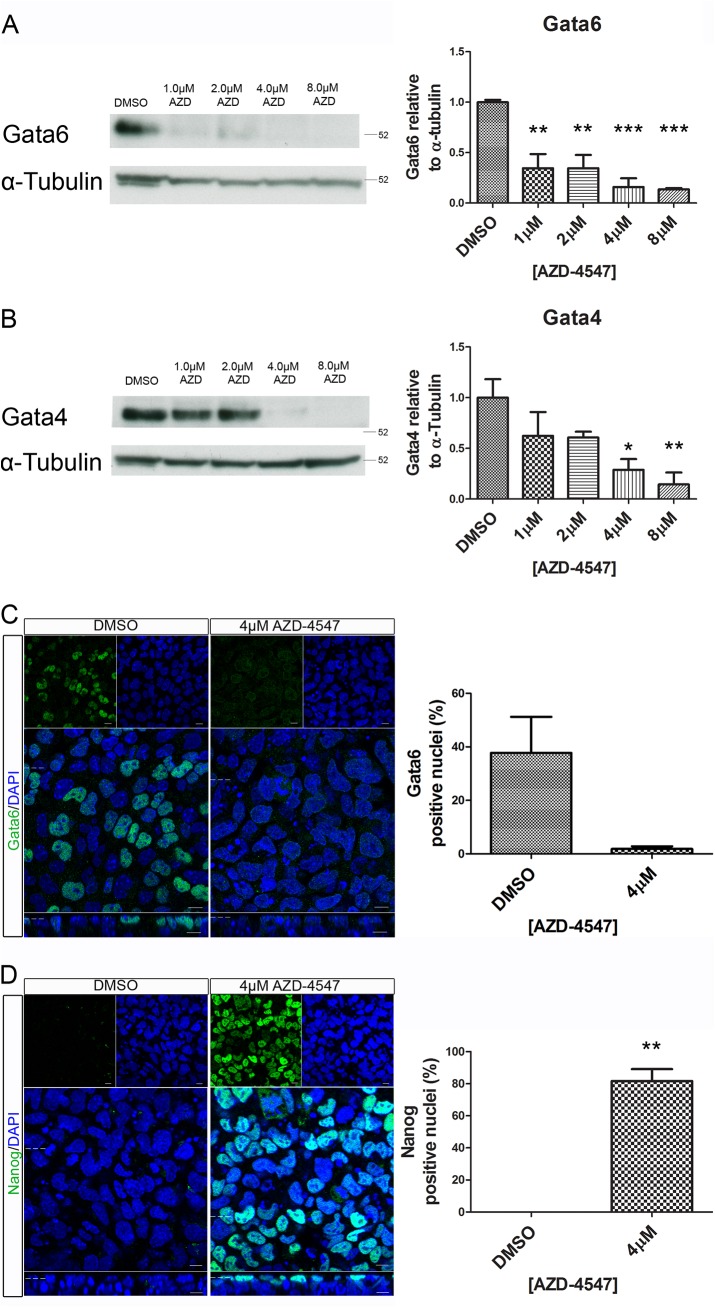
Reduced expression of the primitive endoderm markers Gata4 and Gata6 and increased expression of Nanog was observed in embryoid bodies following inhibitor of the Fgfr. Embryoid bodies were grown in different concentrations of AZD-4547 or 0.08% DMSO for 7 days. Expression levels of (A) Gata6, and (B) Gata4 were analysed using western blotting. A representative blot and quantification from 3 independent experiments is shown for each marker. A dose dependent decrease in expression of both proteins was observed. Statistical analysis is a one-way Anova with a Dunnett’s post-hoc test. Whole-mount immunostaining of (C) Gata6 and (D) Nanog after treatment of embryoid bodies with 4μM AZD-4547 or 0.04% DMSO. A reduction in the percentage of nuclei expressing Gata6 was observed. The percentage of nuclei expressing Nanog increased. A representative image from 3 independent experiments is shown. Scale bars 10μm. Dotted lines represent position that the relevant orthogonal or aerial images were taken. Statistical analysis is a paired t-test. Data is from 3 independent experiments, error bars represent SEM. (* P = 0.1–0.5, ** p = 0.001–0.01, *** p<0.001)

The Western blot for aPkcζ/λ in Fig 9B incorrectly appears as a duplicate of the Fibronectin blot in Fig 9F. Please see the corrected Fig 9 here.

**Fig 9:**
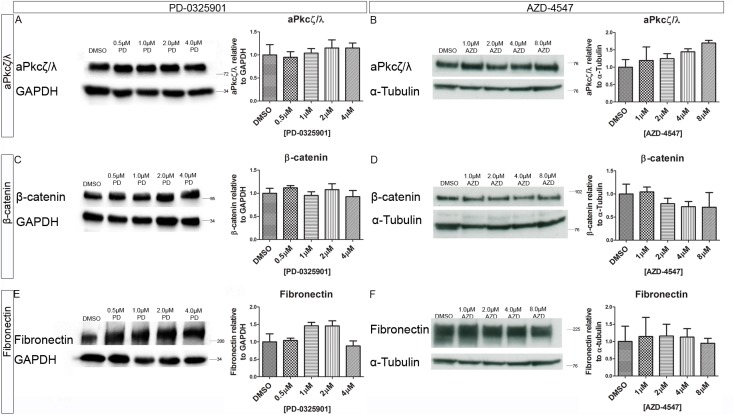
Inhibition Mek or the Fgfr did not significantly alter the expression levels of polarity and junction proteins. Embryoid bodies were grown in different concentration of the (A, C, E), Mek inhibitor PD-0325901 and 0.04% DMSO or the (B, D, F) Fgfr inhibitor AZD-4547 and 0.08% DMSO for 7 days. Expression levels of polarity and junction proteins were assessed using western blotting (A&B) aPkcζ/λ, (C&D) β-catenin, and (E&F) Fibronectin. A representative blot and quantification from 3 independent experiments is shown for each marker. No statistically significant change was seen in any of the markers observed. Statistical analysis is a one-way Anova with a Dunnett’s post-hoc test. Error bars represent SEM. (* P = 0.1–0.5, ** p = 0.001–0.01, *** p<0.001)

The authors confirm that these changes do not alter their findings. Original, uncropped images for Figs 6A (1µM and 2µM), 7D (original confocal files), 9B, 9D and 9F are available as Supporting Information.

## Supporting Information

S1 File Raw images for Figure 6A. (ZIP)

S2 File Raw images for Figure 7D. These require Zeiss LSM Image Browser to view, which is available to freely download from Zeiss.
(ZIP)

S3 File Raw images for Figure 9B, D, and F. (ZIP)

## References

[1] Doughton G, Wei J, Tapon N, Welham MJ, Chalmers AD (2014) Formation of a Polarised Primitive Endoderm Layer in Embryoid Bodies Requires Fgfr/Erk Signalling. PLoS ONE 9(4): e95434 doi:10.1371/journal.pone.0095434 24752320 10.1371/journal.pone.0095434 PMC3994041

