# Supplementary figures and images for: Correction: Formation of a Polarised Primitive Endoderm Layer in Embryoid Bodies Requires Fgfr/Erk Signalling
Source: PLoS One. 2015 Oct 30;10(10):e0141401. doi: 10.1371/journal.pone.0141401 (PMC4627655; doi:10.1371/journal.pone.0141401)

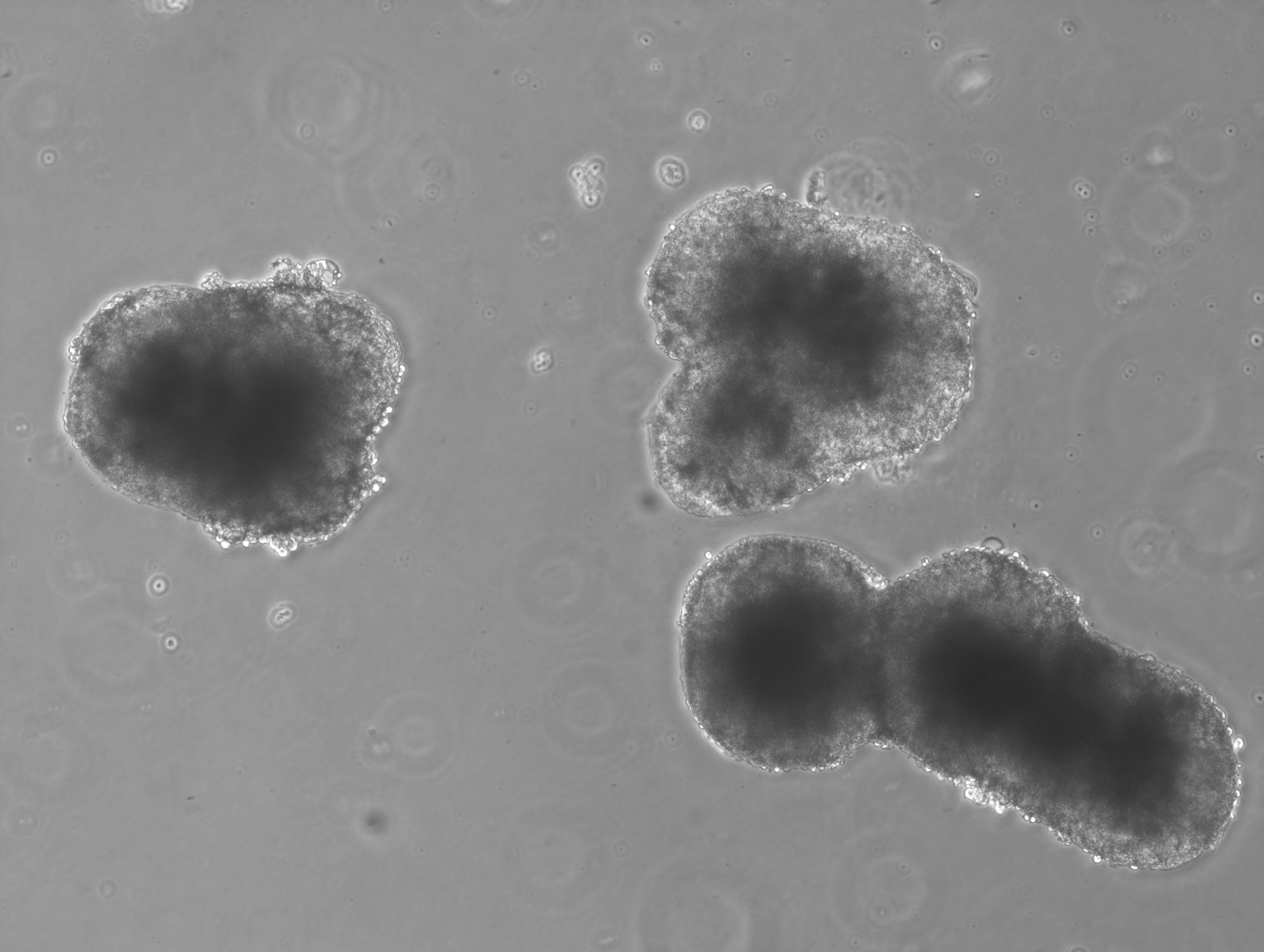

Supplement: S1 File — (ZIP) [file pone.0141401.s001.zip › 1uM130821_003.BMP]

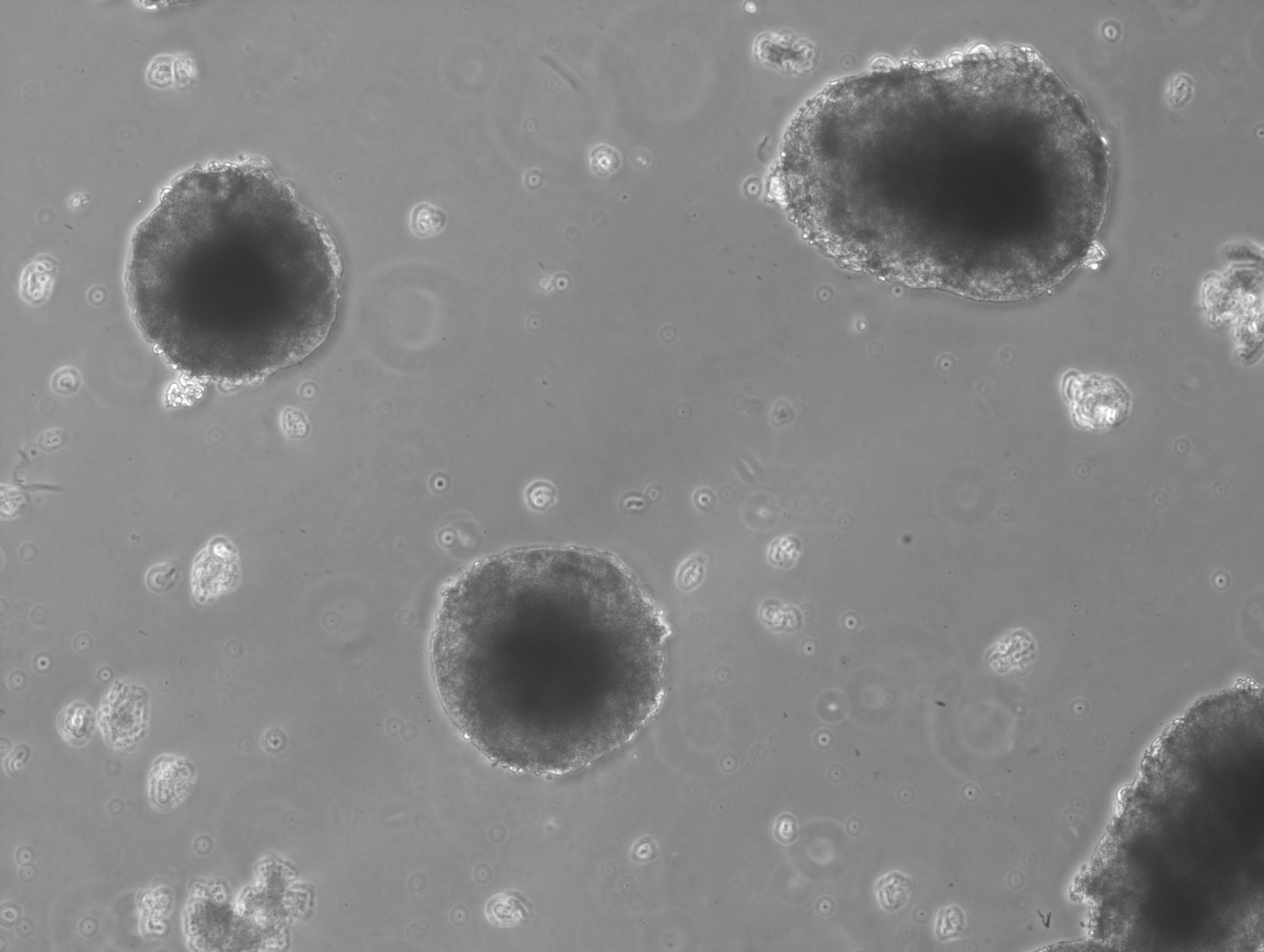

Supplement: S1 File — (ZIP) [file pone.0141401.s001.zip › 2uM130821_002.BMP]

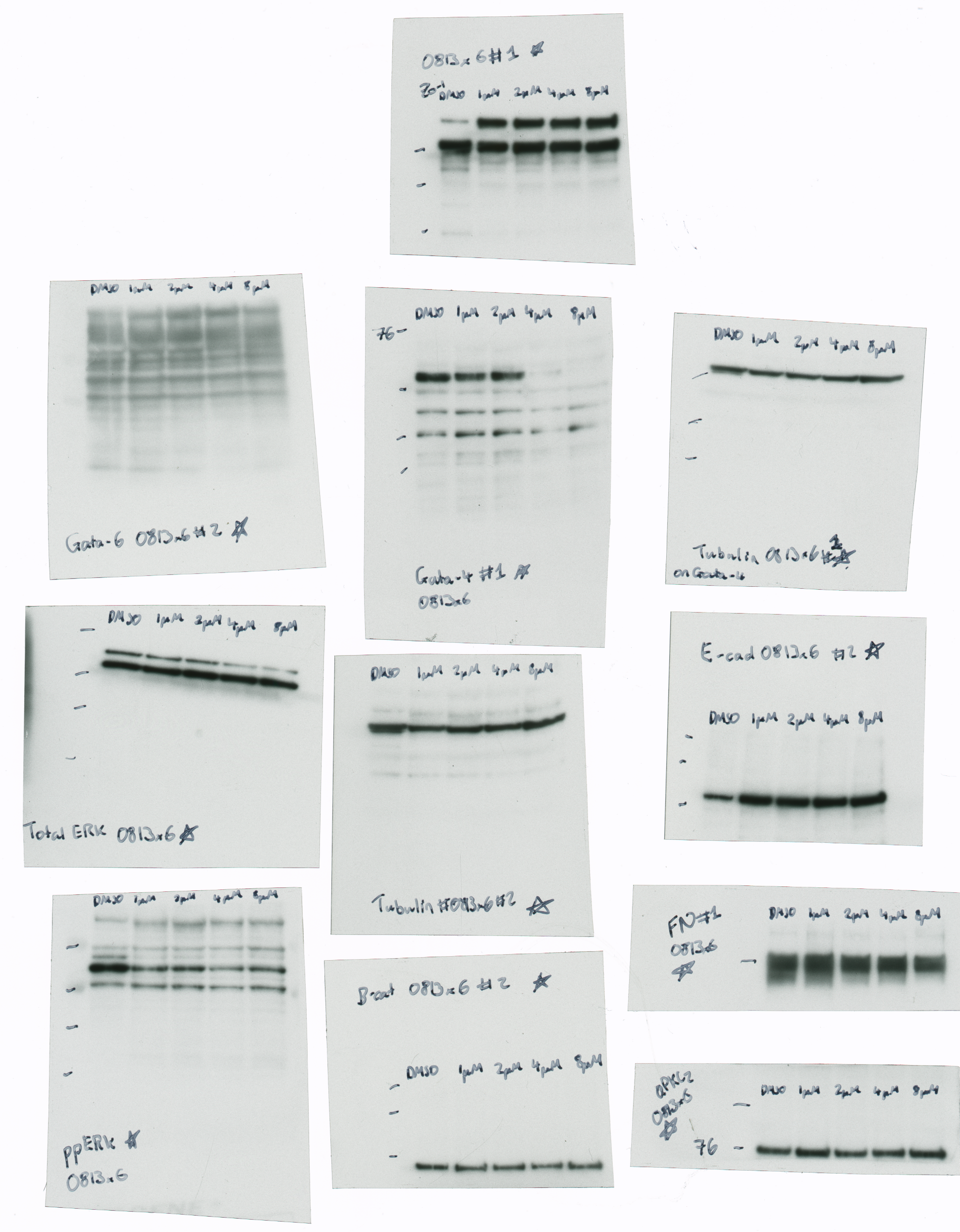

Supplement: S3 File — (ZIP) [file pone.0141401.s003.zip › 0813x6.tif]
